# A Chimera Containing CD4+ and CD8+ T-Cell Epitopes of the *Leishmania donovani* Nucleoside Hydrolase (NH36) Optimizes Cross-Protection against *Leishmania amazonesis* Infection

**DOI:** 10.3389/fimmu.2017.00100

**Published:** 2017-02-23

**Authors:** Marcus Vinícius Alves-Silva, Dirlei Nico, Alexandre Morrot, Marcos Palatnik, Clarisa B. Palatnik-de-Sousa

**Affiliations:** ^1^Laboratório de Biologia e Bioquímica de Leishmania, Departamento de Microbiologia Geral, Instituto de Microbiologia Paulo de Góes, Universidade Federal do Rio de Janeiro, Rio de Janeiro, Rio de Janeiro, Brazil; ^2^Programa de Pós-Graduação em Biotecnologia Vegetal e Bioprocessos, Centro de Ciências da Saúde, Universidade Federal do Rio de Janeiro, Rio de Janeiro, Rio de Janeiro, Brazil; ^3^Laboratório de Imunologia Integrada, Departamento de Imunologia, Instituto de Microbiologia Paulo de Góes, Universidade Federal do Rio de Janeiro, Rio de Janeiro, Rio de Janeiro, Brazil; ^4^Programa de Pós-Graduação em Clínica Médica, Faculdade de Medicina-Hospital Universitario Clementino Fraga Filho, Universidade Federal do Rio de Janeiro, Rio de Janeiro, Rio de Janeiro, Brazil; ^5^Faculdade de Medicina, Instituto de Investigação em Imunologia, Universidade de São Paulo (USP), São Paulo, São Paulo, Brazil

**Keywords:** nucleoside hydrolases, T-cell epitope vaccines, visceral and cutaneous leishmaniasis, *Leishmania amazonensis*, PADRE epitopes

## Abstract

The *Leishmania donovani* nucleoside hydrolase (NH36) and NH A34480 of *Leishmania amazonensis* share 93% of sequence identity. In mice, the NH36 induced protection against visceral leishmaniasis is mediated by a CD4+ T cell response against its C-terminal domain (F3). Besides this CD4+ Th1 response, prevention and cure of *L. amazonensis* infection require also additional CD8+ and regulatory T-cell responses to the NH36 N-terminal (F1 domain). We investigated if mice vaccination with F1 and F3 domains cloned in tandem, in a recombinant chimera, with saponin, optimizes the vaccine efficacy against *L. amazonensis* infection above the levels promoted by the two admixed domains or by each domain independently. The chimera induced the highest IgA, IgG, and IgG2a anti-NH36 antibody, IDR, IFN-γ, and IL-10 responses, while TNF-α was more secreted by mice vaccinated with F3 or all F3-contaning vaccines. Additionally, the chimera and the F1 vaccine also induced the highest proportions of CD4+ and CD8+ T cells secreting IL-2, TNF-α, or IFN-γ alone, TNF-α in combination with IL-2 or IFN-γ, and of CD4+ multifunctional cells secreting IL-2, TNF-α, and IFN-γ. Correlating with the immunological results, the strongest reductions of skin lesions sizes were determined by the admixed domains (80%) and by the chimera (84%), which also promoted the most pronounced and significant reduction of the parasite load (99.8%). Thus, the epitope presentation in a recombinant chimera optimizes immunogenicity and efficacy above the levels induced by the independent or admixed F1 and F3 domains. The multiparameter analysis disclosed that the Th1-CD4+ T helper response induced by the chimera is mainly directed against its FRYPRPKHCHTQVA epitope. Additionally, the YPPEFKTKL epitope of F1 induced the second most important CD4+ T cell response, and, followed by the DVAGIVGVPVAAGCT, FMLQILDFYTKVYE, and ELLAITTVVGNQ sequences, also the most potent CD8+ T cell responses and IL-10 secretion. Remarkably, the YPPEFKTKL epitope shows high amino acid identity with a multipotent PADRE sequence and stimulates simultaneously the CD4+, CD8+ T cell, and a probable T regulatory response. With this approach, we advanced in the design of a NH36 polytope vaccine capable of inducing cross-protection to cutaneous leishmaniasis.

## Introduction

Leishmaniasis is a complex of protozoan vector-borne diseases of recent increased worldwide incidence (1). Clinical manifestations of the disease range from visceral leishmaniasis (VL), the most severe and fatal syndrome with 400,000 new cases/year, characterized by a strong suppression of the CD4+ T-cell response, to tegumentary leishmaniasis, with 7 to 1.2 million new annual cases and variable degrees of T-cell immunity (2). Clinical cases of cutaneous leishmaniasis (CL), the most benign form of tegumentary leishmaniasis, show skin ulcers and a T cell-mediated active immune response, which is often responsible of self-healing or worsening of the disease (2). Mucocutaneous leishmaniasis (MCL), on the other hand, involves an exacerbated immune inflammatory response and lesions of cutaneous and mucosal tissues, while diffuse cutaneous leishmaniasis (DCL), the anergic form of disease, is associated to high *Leishmania*-specific inhibition of the T-cell responses (3). A few patients also develop the borderline disseminated leishmaniasis (4).

Afghanistan, Algeria, Colombia, Brazil, Iran, Syria, Ethiopia, North Sudan, Costa Rica, and Peru are the 10 countries with higher incidence of CL, and together they account for 70–75% of the estimated global occurrence (2). Regarding the ethyological agents of tegumentary leishmaniasis, *Leishmania amazonensis* is a causative agent of CL, MCL, and DCL in Northern, South America, and Brazil (3–7).

Chemotherapy of leishmaniasis is highly toxic, and many cases of resistance or recurrent disease were reported (8–10). Alternatively, vaccine-mediated prevention or cure of CL was assayed with first generation formulations since the 80s, achieving, however, no more than 50% efficacy (8, 11). Only one vaccine based on *L. amazonensis* lysate is licensed at present for immunochemotherapy in Brazil (8).

Since three licensed vaccines against canine VL are available at present (12–14), one feasible approach to induce cross-protection against CL would be to use the vaccine antigens that are conserved in the *Leishmania* genus (15, 16) and already demonstrated to confer protection against VL (12, 17–19), the most immunosuppressive and severe form of the disease.

The *Leishmania donovani* nucleoside hydrolase (NH36) (17) is the main antigen of the Leishmune^®^ vaccine, the first licensed veterinary vaccine against canine VL (12, 18). Leishmune^®^ shows 76–80% vaccine efficacy (18, 19), and its use in endemic areas already promoted the decrease of the canine and the human incidence of VL (12).

Nucleoside hydrolases are enzymes of the DNA metabolism of bacteria, fungi, and protozoa which release exogenous purines or pyrimidines from nucleosides, in microorganisms that are not able to synthetize them, enabling in this way an efficient pathogen replication. They are absent in mammals (20, 21).

Vaccination with the NH of *L. donovani*, NH36, in its native form, protected mice against *L. donovani* infection (22), and in its DNA or recombinant protein forms induced efficacy against mice (17, 23, 24) and dog infections by *Leishmania chagasi* (25), and against mice challenged with *Leishmania mexicana* (23), *Leishmania major* (26), and *L. amazonensis* (27–29), the respective agents of cutaneous and diffuse leishmaniasis. NHs are considered strong phylogenetic markers of the genus *Leishmania* (15, 16), and their amino acid sequences are strongly conserved (29, 30). In fact, the sequence of *L. donovani* NH36 is homologous to the NH sequences of all the studied species of *Leishmania*: *L. major* (95%) (31), *L. chagasi* (99%), *Leishmania infantum* (99%), *L. amazonensis* (93%) (28), *L. mexicana* (93%), *Leishmania braziliensis* (84%), and *Leishmania tropica* (97%) (32).

Therefore, NH36 becomes a good candidate for the development of a cross-protective and universal vaccine against leishmaniasis. Using recombinant generated proteins covering the whole sequence of NH36, and saponin, in previous work, we demonstrated that protection against mice VL is mediated by a CD4+ T cell response against epitopes of the NH36 C-terminal domain (F3) (17). On the other hand, prevention (28) and cure of mice CL (29) caused by *L. amazonensis* are determined by a CD4+-Th1 cell-mediated response toward the F3 protein and a CD8+ and regulatory T-cell responses directed to the N-terminal (F1) domain of NH36, which promoted simultaneous increased secretions of IFN-γ, TNF-α, and IL-10 (29).

Additionally, the F3 vaccine promoted in mice a 36 and 40% respective higher average protection than those generated by the NH36-vaccine against VL, induced by *L. chagasi* (17), and CL, caused by *L. amazonensis* (28). These results confirmed that the use of the domain containing the relevant epitopes enhances the efficacy over that induced by the cognate whole protein (33).

Multisubunit vaccines against leishmanial infection have been shown to be more promising (34). Additionally, the most efficient protection is considered to be determined by diverse T cells that respond to a group of the pathogen-derived epitopes (35). Recent research in immunity to leishmaniasis disclosed the importance of multifunctional CD4+ and CD8+ T cells in the generation of a Th1 response to control infection (34, 36, 37). According to that, it has been suggested that the *in silico* tools should be used to search in the *Leishmania* genome for potential candidates with both CD4+ and CD8+ T cell-stimulating competences. Epitope mapping could then be used to design a polyepitope vaccine that could carry conserved epitopes, which bind to many HLA or MHC allotypes, or which are present in many species of a *Leishmania* genus (34).

Although the whole NH36 protein would be easier to work with, it would be also much less potent. In fact, according to the philosophy of the development of T-epitope vaccines, the whole cognate protein is less potent than the domains that contain the important epitopes. The domains are also more immunogenic than the isolated epitopes. Our aim, in this investigation, was to increase potency and optimize the vaccine. In agreement to that, we were able to show that a combination of these domains in a chimera even enhances the protective efficacy demonstrated for each domain independently. Our results support that the use of the domain containing the relevant epitopes enhances the vaccine efficacy over that induced by the cognate whole protein (33).

In fact, we evaluated if the administration of F1 and F3 domains of NH36 expressed in tandem, as a recombinant chimera, optimizes the immunogenicity and vaccine efficacy against mice infection by *L. amazonensis*, above the levels promoted by the two admixed domains and by each protein administered alone. We additionally identified the most important epitopes of F1 and F3 responsible for the generation of the cellular immune response. In this investigation, we aimed to progress in the development of a universal NH36 T-cell epitope vaccine capable of protecting against the infection by *L. amazonensis*.

## Materials and Methods

### Ethical Statement

This study was carried out in accordance with the recommendations of National Institutes of Health, USA. The protocol was approved by the Comissão de Avaliação da Utilização de Animais em Pesquisa do Centro de Ciência da Saúde (CEUA), Universidad Federal do Rio de Janeiro (CONCEA, Brazil, 01200.001568/2013-87, IMPPG-016).

### Recombinant Antigens Expression and Purification and Epitopes

NH36 is composed of 314 amino acids (Genbank access number AY007193, SwissProt-UniProt access number Q8WQX2-LEIDO). The N-terminal (F1, amino acid sequences 1–103), the central (F2, amino acids 104–198), and the C-terminal (F3, amino acids 199–314) domains were cloned in the pET28 plasmid system, between the restriction sites of *Nco*I and *Xho*I (17). Furthermore, the F1F3 recombinant chimera containing the sequence of the F1 followed by the F3 protein, cloned between the restriction sites of *Nco*I and *Xho*I was obtained with optimized codons in the PET28b expression vector by GenScript (NJ, USA). All the recombinant proteins used in this investigation were expressed with a six His-Tag at their C-terminals.

For protein expression, pET28bNH36, pET28bF1, pET28bF3, or pET28bF1F3-transformed *E. coli* BI21DE3 bacteria were amplified into 2 l of LB culture medium with kanamycin. Expression was induced with 1 mM IPTG (isopropyl-beta-d-thiogalactopyranoside—Fermentas) for 4 h at 37°C, and cultures were centrifuged. The pellets were sonicated and further centrifuged for 20 min under 14,000 rpm at 4°C. The recombinant proteins were more concentrated in the pellets, which were purified by affinity column chromatography with Ni-NTA resin according to the manufacturer’s instructions (Qiagen). Briefly, each pellet was incubated with 10–15 ml urea buffer (8 M urea, 1 M Na_2_HPO_4_, 1 M NaH_2_PO_4_, and 1 M Tris–HCl) pH 8.0, for 2 h, on ice. Then, the suspension was homogenized by successive passages through 20 ml syringes with 1.2 mm × 40 mm needles, until complete dissolution and centrifuged for 15 min at 14,000 rpm at 4°C. The supernatants containing the solubilized proteins were loaded on the Ni-NTA column previously equilibrated with urea buffer. Then, the column was washed with 5 volumes of urea buffer, pH 8.0, for removal of non-specifically bound proteins and with additional 5 volumes of urea buffer, pH 6.4–6.5, for removal of bacterial proteins containing His residues. The recombinant antigens were recovered in 10 volumes of urea buffer pH 4.5, dialyzed overnight against PBS at 4°C, and preserved with 1 mM PMSF and 5% glycerol at −80°C (17). The absence of LPS was confirmed using the LAL QCL-1000 kit (Lonza).

Furthermore, CD4+ T cell epitopes predicted by the Protean Pad program based on the A. Sette algorithm for the H2^d^ haplotype of Balb/c mice (IA^d^ and IE^d^ alleles) (17) and the CD8+ T cell epitopes (H2 L^d^ haplotype), identified by the HLA peptide motif search^1^ and the SYFPEITHI^2^ programs (17) were synthetized by GenScript (NJ, USA). A model of the structure of the NH36 monomer and of the spatial distribution of the predicted epitopes in F1 and F3 is represented in Figure S1 in Supplementary Material.

### Vaccine Efficacy Assay

Groups of BALB/c females (2-month old) were randomized by corporal weight and immunized with three subcutaneous doses of each respective vaccine: F1 (100 μg), F3 (100 μg), F1 (50 μg), and F3 (50 μg) administered as a simple mixture (F1 + F3), and F1F3 chimera (100 or 200 μg). All antigens were formulated with 100 μg of Riedel de Haen saponin (Sigma, St. Louis, MO, USA) in 0.2 ml of 0.9% NaCl saline solution and injected subcutaneously in the back, at weekly intervals (17, 28, 29). Control animals received only saline. Mice were infected subcutaneously in the right hind footpads with 10^6^ stationary phase infective promastigotes of *L. amazonensis* IFLA/BR/67/PH8, 10 days after the last vaccine dose. Briefly, infective parasites were obtained from hamsters footpads in 10% fetal calf serum, 50 U penicillin, and 50 μg streptomycin/ml (Cultilab, Brazil) supplemented Schneider’s Drosophila medium (Sigma). Parasites where then cultured using a 1/5 serial dilution, in 24-well plates, at 26°C, during 3 days. On day 4, 400 μl of promastigote suspensions was amplified through three successive passages in Schneider’s supplemented medium and finally, the stationary-phase parasites were centrifuged, washed twice, suspended in saline solution, counted in a hemocytometer, and used for infection.

The evolution of lesion sizes in footpads was monitored weekly, with a Mitutoyo^®^ pachymeter. Measures of the infected footpads were subtracted from the contra-lateral controls injected only with saline.

At week 12, mice were euthanized with CO_2_ and their parasite load determined by a limiting dilution assay. Briefly, the infected paws were removed under aseptic conditions and washed with Schneider’s medium. Fragments of bones, nails, and skin were chirurgically removed. The remaining tissue was chopped into small pieces and suspended into 1 ml supplemented Schneider’s medium. A 1/5 serial dilution of this suspension was obtained in a 24-well plate that was further incubated at 26°C for 4 days, with daily observation in an inverted microscope. The number of promastigotes present at the last well containing visible parasites was quantified in a hemocytometer.

The anti-NH36 antibody response was assessed in sera, and the intradermal response to leishmanial antigen, and the secretion and intracellular expression of cytokines by antigen-stimulated CD4 and CD8 lymphocytes were studied 1 week after complete vaccination and on week 12 after infection.

### Antibody Assay in Sera

Anti-NH36 antibodies were assayed in the sera of mice, using a standard ELISA assay. Plates were treated with 2 μg NH36 per well in carbonate–bicarbonate buffer pH 9.6, washed with 0.018 M PBS, pH 7.2, 1% non-fat milk, and 0.05% Tween-20 buffer (PBS*), and further incubated with diluted sera samples in PBS* for 1 h at 37°C. Then, plates were washed and treated with peroxidase-conjugated goat anti-mouse-IgA, IgM, IgG1, or IgG2a antibodies (1:4,000) (Southern Biotechnology Associates, Birmingham, AL, USA) or with protein-A-peroxidase (Kirkegaard & Perry Laboratories, Gaithersburg, MD, USA, EEUU) at 1:1,000 dilution in PBS*. Reaction was developed with ortho-phenylenediamine buffer (Sigma), interrupted with 1 N sulfuric acid, and monitored at 492 nm. Results were expressed as the mean of absorbance values of 1/100 diluted sera of each animal, in triplicates, in double-blind tests.

### Intradermal Skin Test (IDR)

Mice were injected in their right front footpad with 0.1 ml 0.9% NaCl saline solution containing the lysate of 10^7^ freeze-thawed stationary-phase *L. amazonensis* infective promastigotes obtained as described in Section “Vaccine Efficacy Assay.” The footpad swallow was monitored with a Mitutoyo apparatus, both before and at 0, 24, and 48 h after lysate injection. Each measure was subtracted from the values of the left front footpads injected with only saline (28).

### Secreted Cytokines Assay

Spleens were excised, and single-cell suspensions were prepared in 1 ml RPMI medium (Sigma, Co) supplemented with 10% fetal calf serum (Nutricell, Campinas, São Paulo, Brazil), 1% l-glutamine, and 5 mM 2-β-mercaptoethanol. Then, cells were counted in a hemocytometer, distributed into a 96-well plate (10^6^/well), and incubated with 5 μg/well NH36, or with no addition, for 72 h *in vitro*, at 37°C with 5% of CO_2_. Culture supernatants were collected and assayed for IFN-γ, TNF-α, and IL-10 using the OptEIA mouse ELISA Set II kits (Becton and Dickinson, BD-Biosciences, USA) according to the manufacturer’s instructions. The sensitivity of the assay was established with a range of 0–1,000 pg/ml for TNF-α and IL-10 and of 0–200 pg/ml for IFN-γ. Reactions were developed using biotinylated anti-cytokine antibodies, streptavidin (SAv-HRP) enzymatic reagent, and TMB (Zymed, USA). Absorbances were monitored at 655 nm.

### Intracellular Cytokine Staining (ICS) and Flow Cytometry

Splenocytes suspended in RPMI medium were distributed into 96-well Costar plates (10^6^/well) and stimulated with 25 μg/ml recombinant NH36 or with no addition, for 24 h at 37°C with 5% CO_2_ according to the results of previous experiments. The intracellular expression of IL-2, TNF-α, and IFN-γ by CD4+ and CD8+ T cells was determined by multiparameter analysis (36, 37) after incubation with 10 μg/ml brefeldin (Sigma) for 4 h at 37°C, 5% CO_2_. After washing with FACS buffer (PBS containing 2% bovine fetal calf, 0.1% sodium azide), splenocytes were labeled for 20 min at 4°C, in the dark with rat anti-mouse-CD4FITC (clone GK1.5) and -CD8FITC (clone 53-6.7) monoclonal antibodies (R&D systems, Inc.). Cells were then fixed with 4% para-formaldehyde, washed and treated with FACS buffer containing 0.5% saponin (Sigma), and further stained with IFN-γAPC, IL-2-PerCP-Cy5.5, and TNF-αPE monoclonal antibodies (BD-Pharmingen). For ICS, gating for CD4+ ad CD8+ T cells was performed, and 100,000 events were acquired in a Becton Dickinson FACScalibur. Data were analyzed using the FlowJo program (Tree Star, USA).

### Epitope Assays

Splenocytes of mice vaccinated with the chimera (100 μg/dose) or saline solution and further challenged with 10^6^ promastigotes of *L. amazonensis* were incubated *in vitro* with 25 μg/ml of NH36, F1F3 chimera, each one of the CD4 predicted epitopes of F1 (ELLAITTVVGNQ and DVAGIVGVPVAAGCT) and F3 domains (FMLQILDFYTKVYE, FRYPRPKHCCHTQVA, and KFWCLVIDALKRIG), with the highest scored CD8 predicted epitope of the F1 protein (YPPEKTKL), or with the mixture of all the epitopes, at week 11 after infection. The epitope-specific cellular immune response was studied through the analysis of the cytokine secretion to supernatants and by multiparameter cytometry, as described above.

### Statistical Methods

Means were compared by the Kruskal–Wallis and Mann–Whitney methods. Spearman’s two-tailed correlation test was used for correlation analysis. Furthermore, the slope of the curves of variation of lesion sizes along the time was calculated as the best-fit values (GraphPad Prism6 software). All experiments were performed at least twice, and the indicated error bars are based on the SE.

## Results

### The F1F3 Chimera Optimizes the Antibody Response

We investigated, for all variables, if the vaccine containing the mixture of the F1 and F3 domains (F1 + F3) was more potent than the F1 and F3 vaccines independently. Our strategy also involved the use of the chimera at the dosage of 100 μg, in order to make a fair comparison with the simple addition of the two domains (F1 + F3), since both vaccines are composed of approximately 50 μg of each F1 and F3 proteins. Additionally, we also used the chimera at the dosage of 200 μg, to disclose any potential dose–response effect of increased efficacy determined by a higher dose of the antigen.

**Figure 1:**
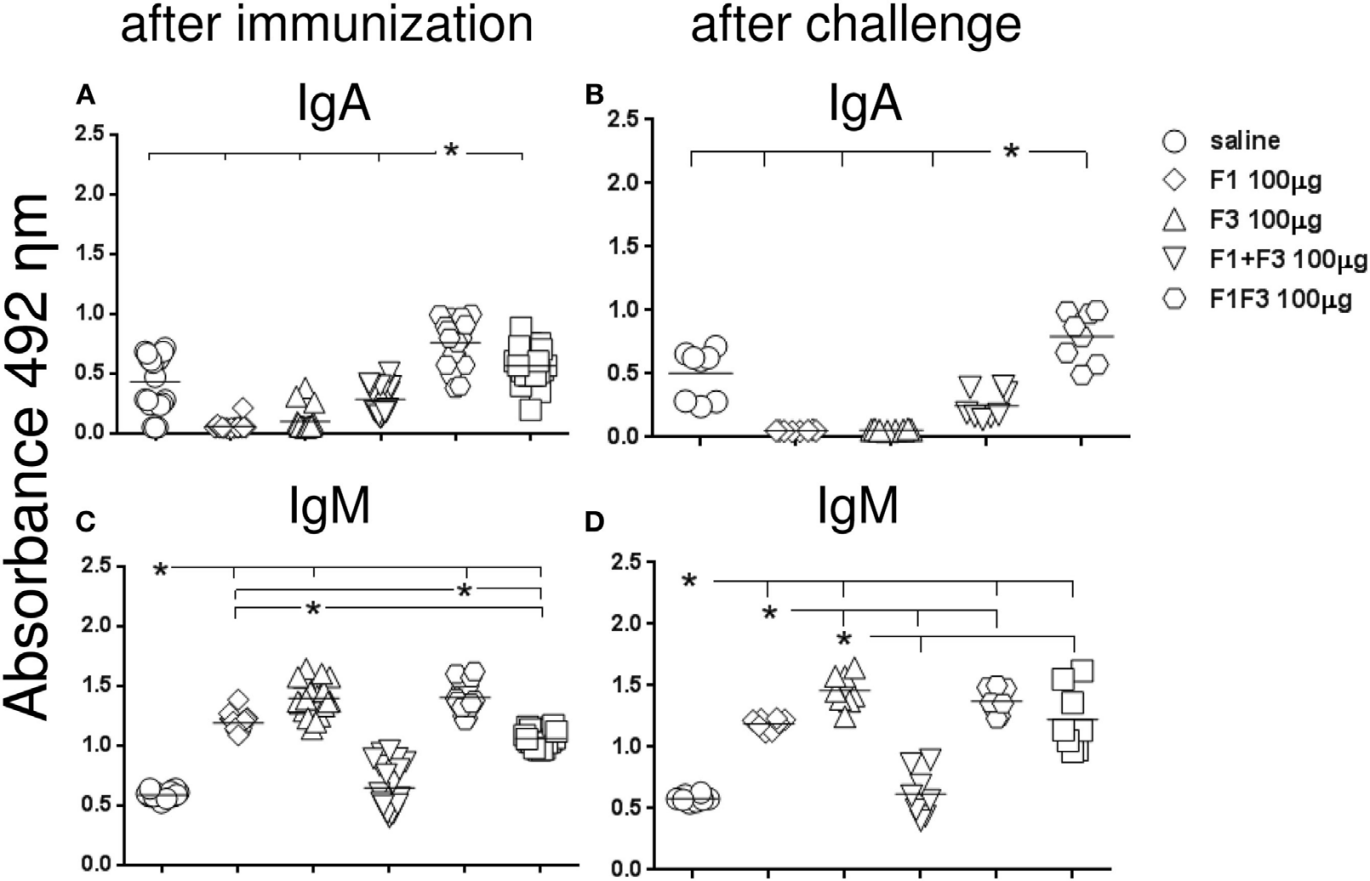
**Chimera enhances the IgA and, together with F3, the IgM anti-NH36 antibody response**. BALB/c mice were immunized with three subcutaneous doses of each respective vaccine: F1 (100 μg), F3 (100 μg), F1 (50 μg), and F3 (50 μg) administered as a simple mixture (F1 + F3), and F1F3 chimera (100 or 200 μg), all formulated in 100 μg of saponin. Anti-NH36 antibodies were measured by an ELISA assay in the sera of mice after vaccination **(A,C)** and on week 12 after infection with *Leishmania amazonensis* **(B,D)**. Data are means and individual results of two independent experiments, each one with 8–10 animals per treatment. The chimera vaccine induced the highest IgA response **(A,B)**. The IgM antibodies were equally enhanced by the F3 and the chimera vaccines before challenge and by the F3 vaccine after challenge **(C,D)**.

**Figure 2:**
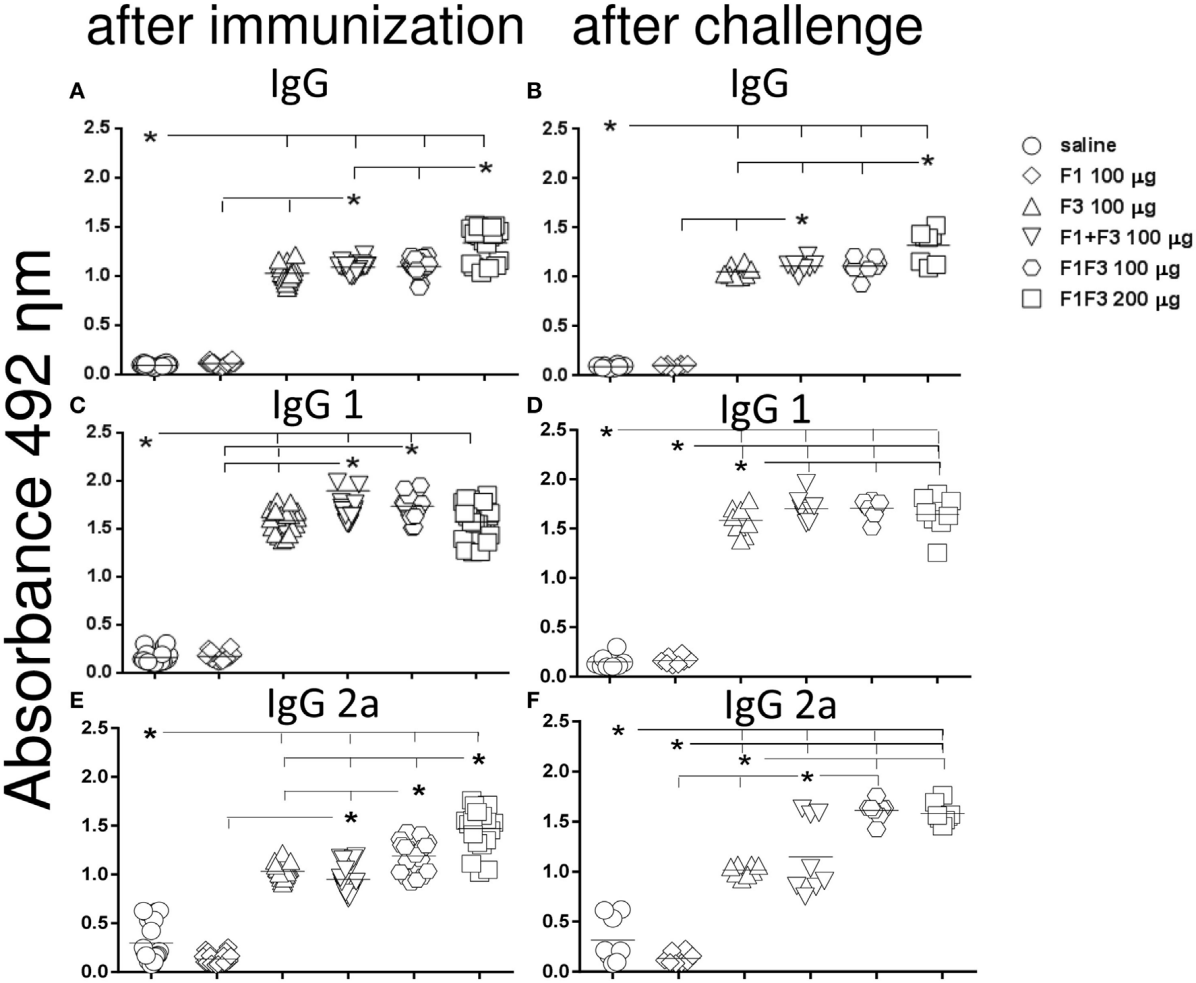
**Chimera increased the IgG, IgG1, and IgG2a antibody levels**. Anti-NH36 antibodies were measured by an ELISA assay in the sera of mice vaccinated with F1, F3, the addition of F1 and F3, or the F1F3 chimera in formulation with saponin and after infection with *Leishmania amazonensis*. Data are means and individual results of two independent experiments, each one with 8–10 animals per treatment. The F1 protein alone did not induce any increase in IgG, IgG1, or IgG2a antibodies while the F3 did **(A–F)**. The mixture of domains enhanced the IgG, IgG1, and IgG2a antibody responses above the F3 and F1 proteins **(A–D,F)**. The chimera, however, induced the strongest IgG and IgG2a anti-NH36 responses **(A–C,E,F)** being, at the dosage of 100 μg, more potent that the domain mixture **(E,F)**, and at the dosage of 200 μg, stronger than at the dosage of 100 μg **(A,B,E)**.

Initially, we compared the antibody response generated by the vaccines, both after complete immunization and after challenge by *L. amazonensis* (Figures 1 and 2). In contrast to what observed for the IgM antibodies (Figures 1C,D), the F1 protein alone did not induce any anti-NH36 antibodies of the IgG class or subclasses. The F3 vaccine on the other hand enhanced the IgM and all IgG antibodies against NH36 above the saline controls (*p* < 0.0001 for all comparisons) (Figures 1C,D and 2A–F). The IgM antibodies were equally enhanced by the F3 and the chimera vaccines before challenge and by the F3 vaccine after challenge (Figure 1).

Furthermore, the admixed domains induced more IgG, IgG1, and IgG2a antibodies than the F3 or the F1 vaccines did independently (*p* < 0.007 for both) indicating that the administration of both proteins simultaneously potentiates the effect (Figures 1A,B and 2).

Remarkably, the chimera vaccine optimized the antibody response by inducing the highest IgA, IgG, and IgG2a anti-NH36 responses (Figures 1A,B and 2A–C,E,F). In fact, the dose of 200 μg determined additional increases of 19%, in the absorbency values, above those of the F1 + F3 and chimera (100 μg) vaccines (*p* < 0.0001 for both) revealing maximal optimization of the IgG response (Figure 2A). The 200 μg F1F3 chimera was also the strongest formulation for the IgG class after challenge (*p* < 0.0148 for all comparisons) (Figure 2B) while the admixed proteins (F1 + F3) were no longer superior to the F3 vaccine.

Noteworthy and in agreement with what described for the IgG response, after immunization, the chimeras induced the most potent and optimized IgG2a response (Figure 2E). The dosage of 100 μg exhibited an increase of 20% (*p* < 0.0001) in the IgG2a antibody titers, above the F1 + F3 vaccine, indicating that the presentation of the epitopes in tandem increases the efficacy. Additionally, and similar to what described for IgG (Figure 1A), the maximal IgG2a antibody response was induced by the 200 μg chimera vaccine, which promoted a 19% higher IgG2a response than the same vaccine at 100 μg dosage (*p* < 0.0001), indicating a dose–response effect (Figure 2E). Optimization of the IgG2a antibody response was also detected as a result of the impact of *L. amazonensis* infection when the two chimera-vaccine dosages showed a 28% increased potency above the F1 + F3 vaccine (*p* < 0.007 for the two comparisons) (Figure 2F).

For the IgG1 subtype, the admixed domains were as potent as the chimera (100 μg) (Figures 2C,D).

We concluded that the presentation of the F1 and F3 proteins in a recombinant chimera determined a stronger effect than the simultaneous delivery of both independent domains, for the induction of IgA, IgG, and IgG2a anti-NH36 antibodies. An increasing dose–response was additionally detected for the chimera in the IgG (before and after challenge) and IgG2a (before challenge) antibodies. In contrast, the chimera at the dosage of 100 μg was even more potent than at 200 μg, for the IgA and IgM (before and after challenge) and IgG1 (after immunization) antibodies, and was as strong as at 200 μg dosage, for the IgG1 and IgG2a (after challenge) response. These results suggest that the optimization effect is more related to the concomitant presentation of the F1 and F3 epitopes in tandem, rather than to an increased antigen concentration.

### The F1F3 Chimera Optimizes the Intradermal Response to *L. amazonensis* Antigen

**Figure 3:**
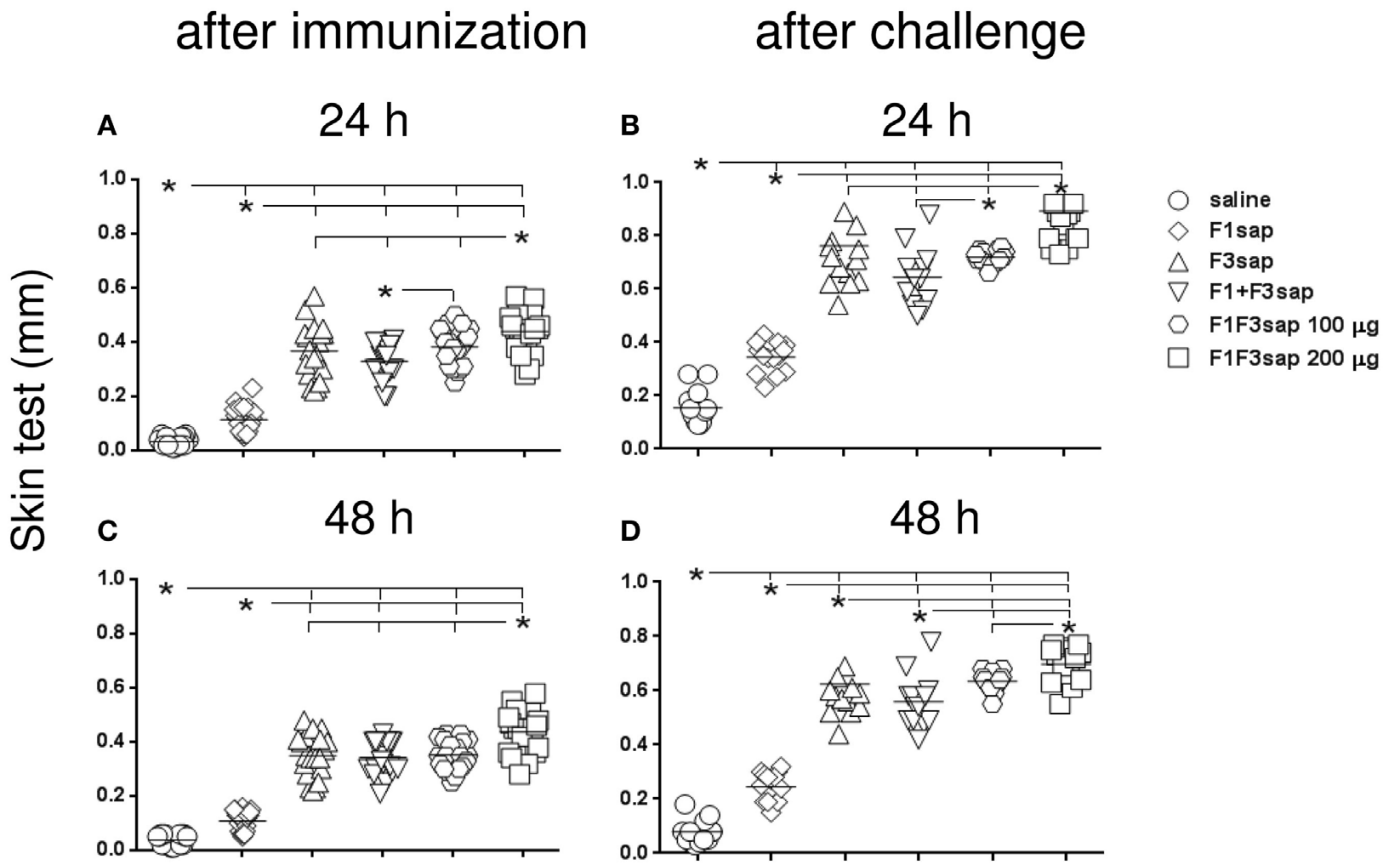
**Chimera optimizes the intradermal response against *Leishmania amazonensis* antigen**. The IDR to the lysate of *L. amazonensis* infective promastigotes was measured in mice vaccinated with F1, F3, the addition of F1 and F3 proteins, or the F1F3 chimera, in formulation with saponin, after vaccination **(A,C)** and on week 12 after challenge with *L. amazonensis* **(B,D)**, at 24 and 48 h after antigen injection. Data are means and individual results of two independent experiments, each one with 8–10 animals per treatment. All formulations were more potent than the saline controls and the F1 vaccine **(A–D)**. The chimera, at 100 μg dosage, was more potent than the addition of domains **(A,B,D)**, and the F3 vaccine **(D)**, but the dosage of 200 μg was the strongest formulation **(A–D)**. After challenge **(B,D)** and 48 h after antigen injection **(C,D)**, all vaccines enhanced the skin tests.

The induction a cellular immune response was initially assessed by IDR. At all times, all formulations developed more potent IDR reactions that the saline controls and the F1 vaccine (*p* < 0.0001 for all comparisons) (Figures 3A–D). Maximal skin tests were achieved in mice vaccinated with the chimera (Figure 3).

Although the chimera at 100 μg dosage was more potent than the admixed proteins (Figures 3A,B,D), and the F3 vaccine (Figure 3D), the dosage of 200 μg was the strongest formulation, which induced the largest skin tests (Figures 3A–D). In fact, at the dosage of 100 μg, the chimera was already 13% more potent than the addition of F1 + F3 proteins (*p* < 0.0232) disclosing the optimization effect that was due to the presentation of the epitopes in tandem. The additional 13% (*p* < 0.0263) enhancement generated by the 200 μg dosage disclosed a dose–response effect (Figure 3A).

After challenge, and in contrast to what described for antibodies, all the vaccines induced significant increases of skin tests (*p* < 0.0001). Actually, after infection, the skin tests of mice vaccinated with the F3, F1 + F3, 100, and 200 μg chimera vaccines were 47, 52, 53, and 51% larger than before challenge, respectively (*p* < 0.0001, for all comparisons) (Figures 3A,B). At 48 h after antigen injection also, respective IDR increases of 44, 38, 44, and 38% were also observed for the F3, F1 + F3, 100, and 200 μg chimera vaccines (*p* < 0.0001 for all comparisons) (Figures 3C,D).

Our results disclosed the chimera at the 200 μg dosage as the strongest formulation. However, at the dosage of 100 μg, the chimera was already capable of inducing the most pronounced enhancement of skin tests after infection, being even more efficacious that the admixed domains. These results confirm the chimera capability for optimization of vaccine efficacy and suggest that efficacy could be even enhanced by using increased chimera dosages (Figures 3A–D).

The IDR response after immunization is highly correlated to the IgG (*p* < 0.0001, *R* = 0.7552, *R*^2^ = 0.5703) and the IgG2a (*p* < 0.0001, *R* = 0.8839, *R*^2^ = 0.7813) antibody responses. Also, after infection, the IgG (*p* < 0.0001, *R* = 0.7009, *R*^2^ = 0.4912) and IgG2a antibody levels (*p* < 0.0001, *R* = 0.8364, *R*^2^ = 0.6995) were correlated to the IDR results.

### Secretion of IFN-γ, TNF-α, and IL-10 Increased in Response to the F1F3 Chimera

**Figure 4:**
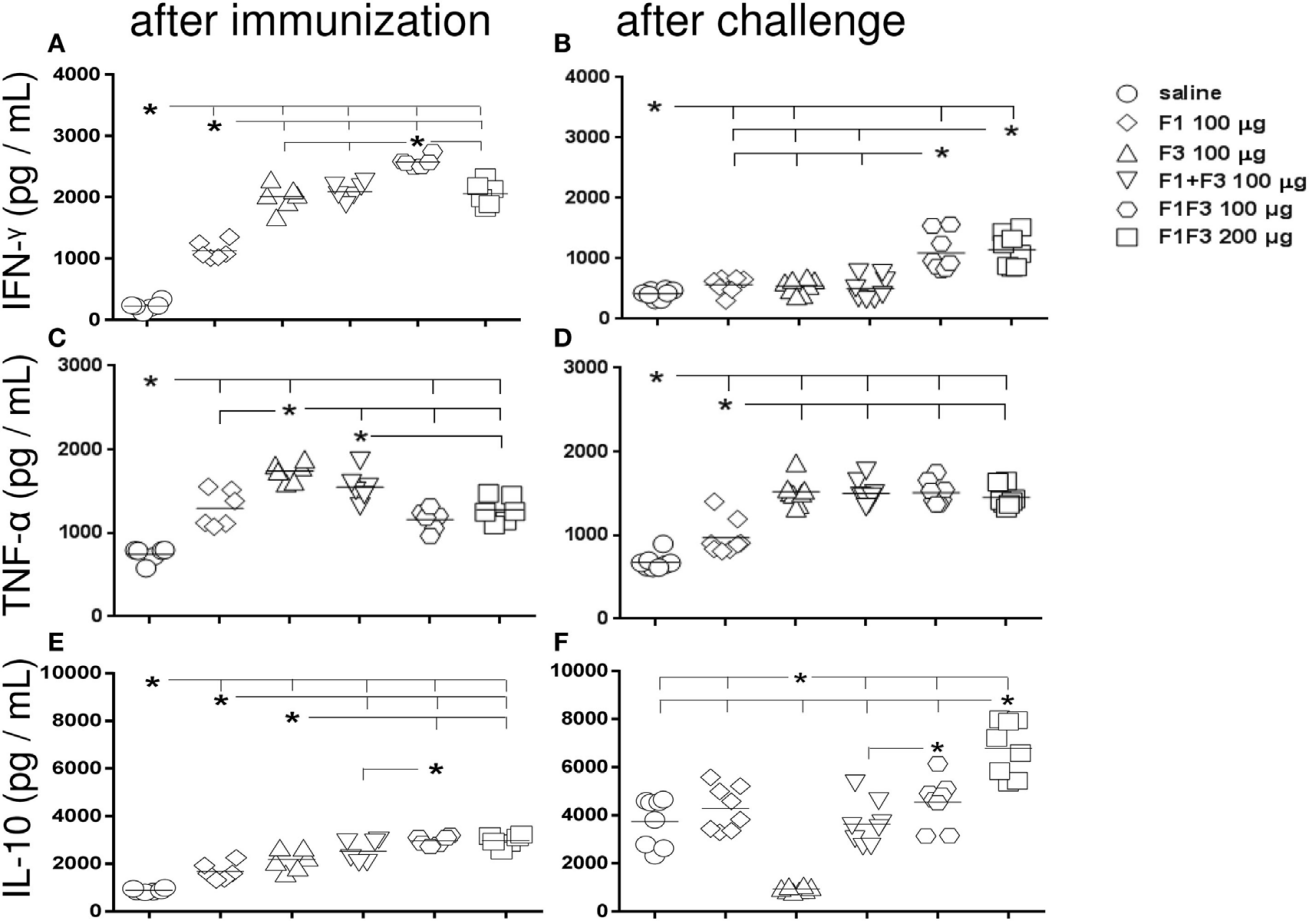
**Chimera potentiates the secretion of IFN-γ and IL-10 while TNF-α is generated in response to all F3-containing vaccines**. Secretions of IFN-γ **(A,B)**, TNF-α **(C,D)**, and IL-10 **(E,F)**, as measured by an ELISA assay in the supernatant of splenocytes, both after immunization with F1, F3, the addition of F1 and F3 proteins, or the F1F3 chimera in formulation with saponin, and after challenge with *Leishmania amazonensis*, are expressed in picograms per milliliter. Data are means and individual results of two independent experiments, each one with 8–10 animals per treatment. After immunization, the F3-containing vaccines secreted more IFN-γ than the F1 vaccine. The chimera at 100 μg dosage was stronger than the admixed proteins and the 200 μg dosage **(A)**. After infection, the two dosages of the chimera were equally potent **(B)** above the F1 and F3 vaccines independently or formulated together. However, the IFN-γ levels after challenge were lower **(B)**. TNF-α was initially more secreted by mice vaccinated with F3 than with F1 **(C)** and by all vaccines containing the F3 domain, after challenge **(D)**. The chimeras or the admixed proteins enhanced IL-10 secretion after immunization, above the F1 or F3 vaccines **(E)**. After challenge, IL-10 secretion was null in F3-vaccinated mice, but it was increased by the other vaccines with maximal performance achieved by the chimera (200 μg) **(F)**. A dose–response increase in IFN-γ **(B)** and IL-10 **(F)** secretion was observed with the increment of the chimera dosage.

After immunization, all vaccines increased the IFN-γ secretion to PBMC supernatants above the levels induced by the saline controls (*p* < 0.0022), indicating the triggering of a Th1-immune response. Mice vaccinated with F3-containing vaccines secreted more IFN-γ than those treated only with the F1 protein (*p* < 0.0022). The main performance was, however, determined by the chimera at 100 μg dosage, which was 19% stronger than the admixed domains and 20% more potent than the 200 μg dosage (*p* < 0.0022 for both comparisons) (Figure 4A). After infection, and as observed for skin tests and IgG2a antibodies, the two dosages of the chimera showed codominance of the IFN-γ secretion (Figure 4B) above the F1 and F3 vaccines independently or formulated together (*p* < 0.0002 for all comparisons). IFN-γ levels induced by the chimeras after challenge were 56–44% lower (Figure 4B).

As observed for IFN-γ and also indicating the rise of a Th1 response, TNF-α was initially more secreted by mice vaccinated with F3 than with F1 (*p* < 0.0022) (Figure 4C) and by all vaccines containing the F3 domain, including the chimeras, after challenge (Figure 4D).

Additionally and suggesting a potential regulatory immune response, mice vaccinated with the chimeras or with the admixed proteins secreted more IL-10 after immunization than mice vaccinated with F1 or F3 proteins alone (*p* < 0.015 for all comparisons) (Figure 4E). Noteworthy, IL-10 secretion was higher in mice vaccinated with 100 μg of the chimera than with the F1 + F3 addition, both before and after challenge (Figures 4E,F).

Remarkably, after challenge, however, IL-10 secretion was null only in F3-vaccinated mice while it was increased by all vaccines containing F1 and achieved the maximal values in the 200 μg chimera vaccine (Figure 4F). In fact, after infection, the IL-10 levels were 2.3 times reduced (*p* < 0.0007) in F3-vaccinated mice, while increases of 2.6 (*p* < 0.0007), 1.4 (*p* < 0.0200), 1.5 (*p* < 0.0023), and 2.3 times (*p* < 0.0007) were detected in mice vaccinated with the F1, F1 + F3, and the chimera vaccine at 100 and 200 μg dosage, respectively (Figures 4E,F).

Lastly, a dose–response increase in IFN-γ and IL-10 secretion was observed with the increment of the chimera dosage to 200 μg.

### The F1F3 Chimera Induces the Highest Proportions of CD4+ and CD8+ T Cells Secreting One, Any Combination of Two and Three Cytokines

The effector function and quality of the T cell immune response were assessed after infection, by multiparameter-flow cytometry analysis.

**Figure 5:**
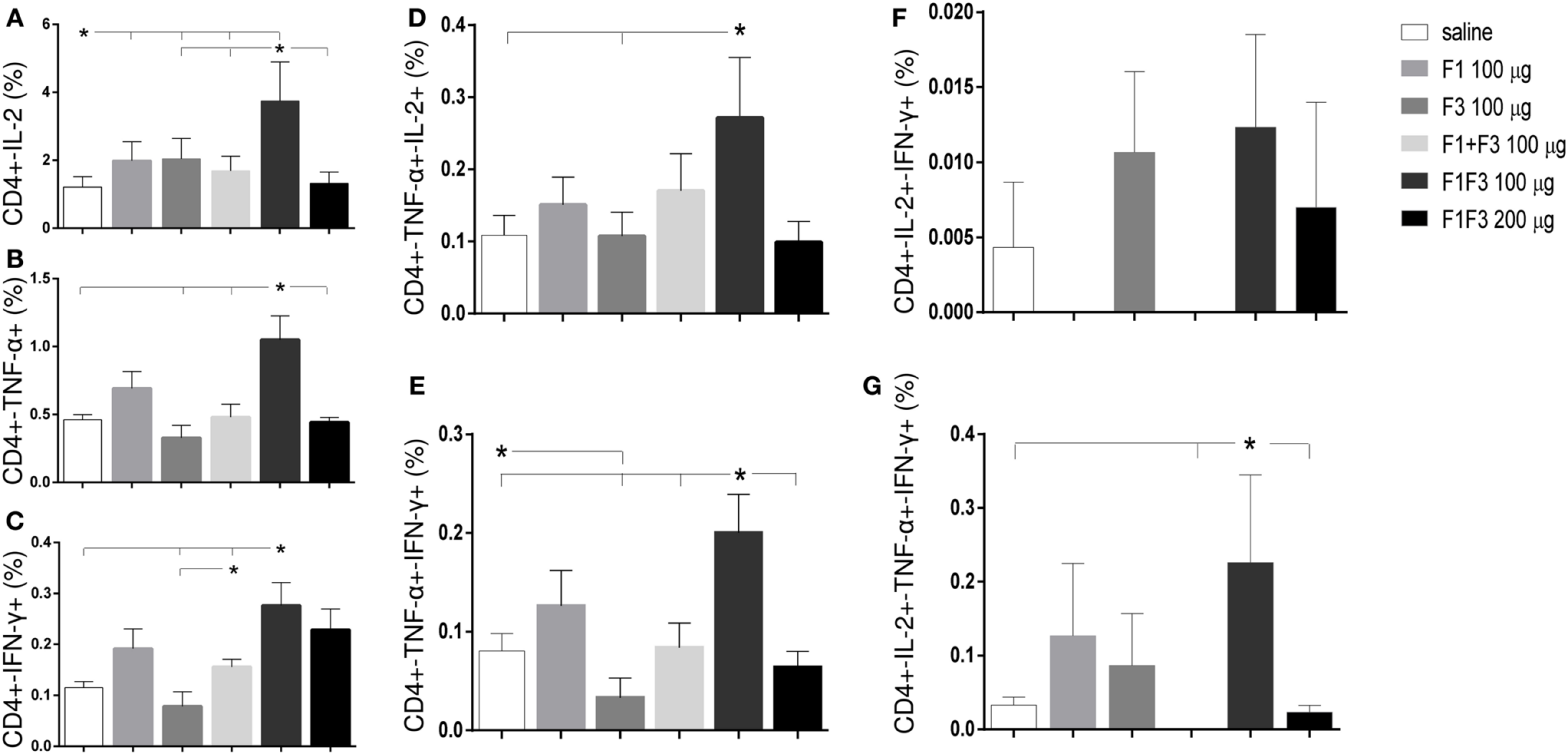
**Chimera and F1 vaccines promote the highest proportions of CD4+ T cells expressing one cytokine, all combinations of two cytokines, or three cytokines after *in vitro* incubation with NH36**. The multifunctional analysis discloses the magnitude of the CD4+ T-cell response of mice immunized and challenged with *Leishmania amazonensis*, by describing the contribution of the frequencies of CD4+ lymphocytes expressing IL-2 **(A)**, TNF-α **(B)**, IFN-γ **(C)**, IL-2/TNF-α **(D)**, TNF-α/IFN-γ **(E)**, IL-2/IFN-γ TNF-α/IFN-γ **(F)**, and IL-2/TNF-α/IFN-γ TNF-α/IFN-γ **(G)** in response to NH36. Bars represent means + SE of two independent experiments, each one with 8–10 animals per treatment. The chimera vaccine at 100 μg/dose induced the highest proportions of all types of CD4+-cytokine secreting T cells **(A–E,G)**. It was stronger than the 200 μg dosage and not significantly different from the F1 vaccine **(A–C,E,G)** for all CD4 T cells subtypes, except for CD4+-TNF-α+-IL-2+ **(C,D)**. Only in the case of CD4+-IL-2+-IFN-γ+ T cells, the differences between the vaccines were no significant **(F)**.

The chimera vaccine at 100 μg/dose induced the highest proportions of all types of CD4+-cytokine secreting T cells (Figure 5). In fact, the 100 μg/dose chimera was more potent than the F3 (*p* < 0.0469) and F1 + F3 vaccines (*p* < 0.0391) inducing elevated proportions of CD4+ T cells secreting IL-2, TNF-α, or IFN-γ alone (Figures 5A–C), TNF-α in combination with IL-2 or IFN-γ (Figures 5D,E) and multifunctional cells secreting IL-2, TNF-α, and IFN-γ simultaneously (Figure 5G). Additionally, the 100 μg dose was stronger than the 200 μg dosage (*p* < 0.0391) and not significantly different than the F1 vaccine (Figures 5A–C,E,G) for all CD4 T cells subtypes, except for CD4+-TNF-α+-IL-2+ (Figures 5C,D). Our results indicate the presence of important CD4+ epitopes in F1 (Figures 5A–E,G) and suggest that the presentation of the epitopes in tandem by the chimera represents the best approach for optimization of the Th1 response. Only in the case of CD4+-IL-2+-IFN-γ+ T cells, the differences between the vaccines were no significant (Figure 5F).

**Figure 6:**
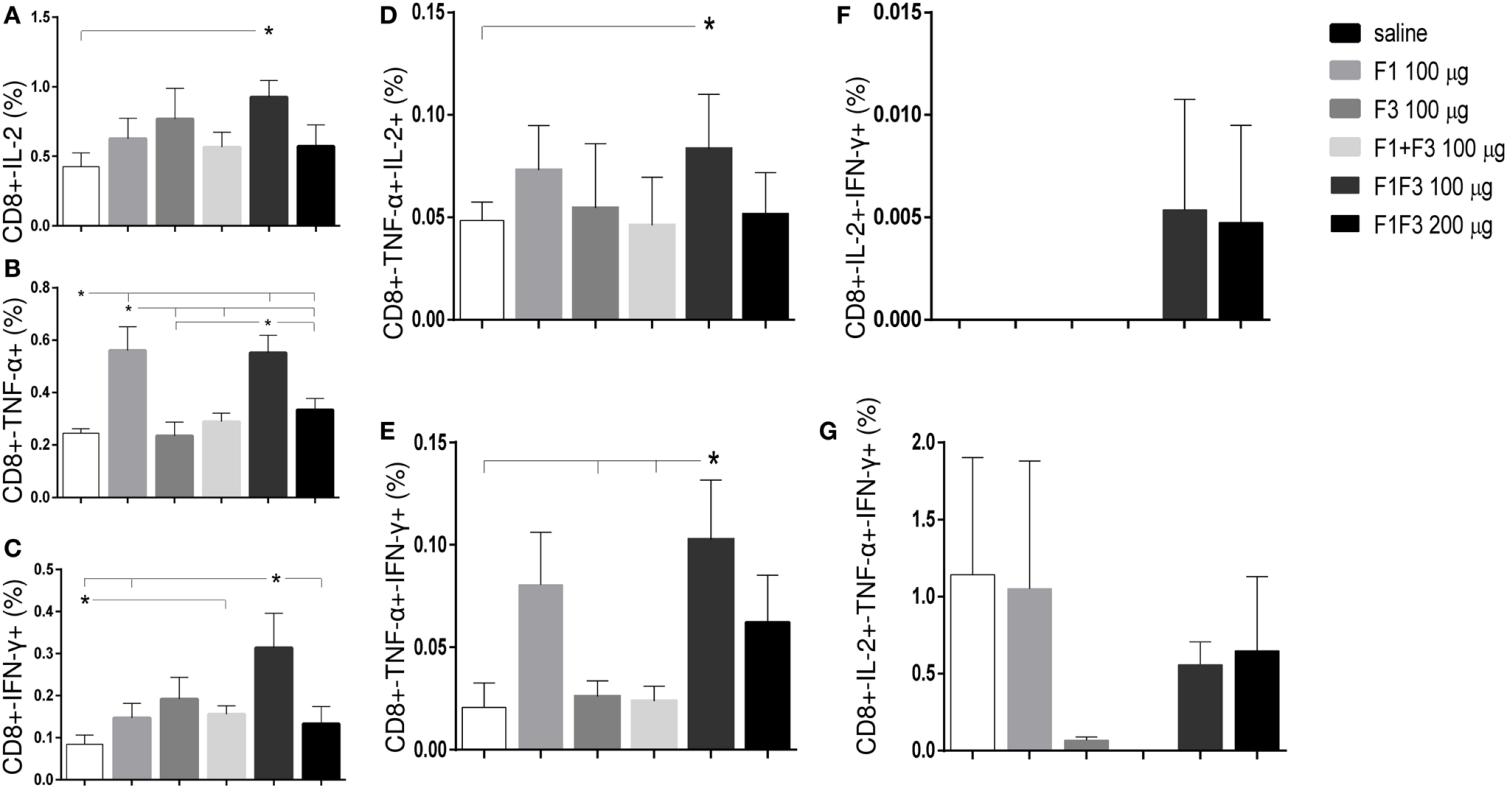
**Chimera and F1 vaccines generate the highest proportions of CD8+ T cells expressing one cytokine, all combinations of two cytokines, or three cytokines after *in vitro* incubation with NH36**. The magnitude of the cytotoxic T-cell response is revealed by the cytometry-multifunctional analysis, which disclosed the frequencies of CD8+ lymphocytes expressing IL-2 **(A)**, TNF-α **(B)**, IFN-γ **(C)**, IL-2/TNF-α **(D)**, TNF-α/IFN-γ **(E)**, IL-2/IFN-γ TNF-α/IFN-γ **(F)**, and IL-2/TNF-α/IFN-γ TNF-α/IFN-γ **(G)** in response to NH36, of mice vaccinated with F1, F3, the addition of F1 and F3, or the F1F3 chimera and challenged with *Leishmania amazonensis*. Bars represent means + SE of two independent experiments, each one with 8–10 animals per treatment. The admixed proteins were not capable of inducing higher CD8+ T cell proportions than the F1 or F3 vaccines. The chimera vaccine (100 μg) promoted the highest proportions of CD8+ T cell expressing all combination of cytokines **(A–E)** except for the CD8+ multifunctional T cells **(G)** and was more potent than at 200 μg for the increase of the proportions of CD8+-TNF-α+ **(B)** and CD8+-IFN-γ+ secreting T cells **(C)**. The 100 μg chimera vaccine was also stronger than the F3 vaccine in CD8+-TNF-α+-IFN-γ T cells and as potent as the F1 vaccine in induction of most types of CD8+-cytokine secreting T cells **(A,B,D,E)**.

Regarding the cytotoxic response (Figure 6), the admixed proteins were not capable of inducing higher CD8+ T cell proportions than the F1 or F3 domains independently (Figure 6). Furthermore, as described for CD4+ T cells (Figure 5), the chimera vaccine, at the dosage of 100 μg, also promoted the highest proportions of CD8+ T cell expressing IL-2, TNF-α, or IFN-γ (Figures 6A–C) and TNF-α, in combination with IL-2 or IFN-γ (Figures 6D,E). This did not occur for the CD8+ multifunctional T cells which, in contrast to what observed for CD4+ T cells, showed no significant differences between treatments (Figures 6F,G).

At 100 μg dosage, the chimera was more potent than at 200 μg for the increase of the proportions of CD8+-TNF-α+ (*p* < 0.0078) and CD8+-IFN-γ+ secreting T cells (*p* < 0.0078). Additionally, and similar to what described for CD4+ T cells (Figure 5), the 100 μg chimera vaccine was stronger than the F3 vaccine in CD8+-TNF-α+-IFN-γ T cells and as potent as the F1 vaccine in induction of most types of CD8+-cytokine secreting T cells (Figures 6A,B,D,E), except for cells secreting only IFN-γ (Figure 6C). Our results also indicate that important epitopes for CD8+ T cells are located in the F1 domain.

The multifunctional flow cytometry confirmed that in order to optimize the antigen presentation to T cells, the exposure of the F1 and F3 domains cloned in tandem is needed. The simple addition of the two domains was not effective in triggering the cellular immune response. We conclude that the chimera at the dosage of 100 μg generated the strongest protection against the parasite challenge but was not significantly different from the F1 vaccine.

### Vaccine Efficacy Is Optimized by the F1F3 Chimera Formulation

**Figure 7:**
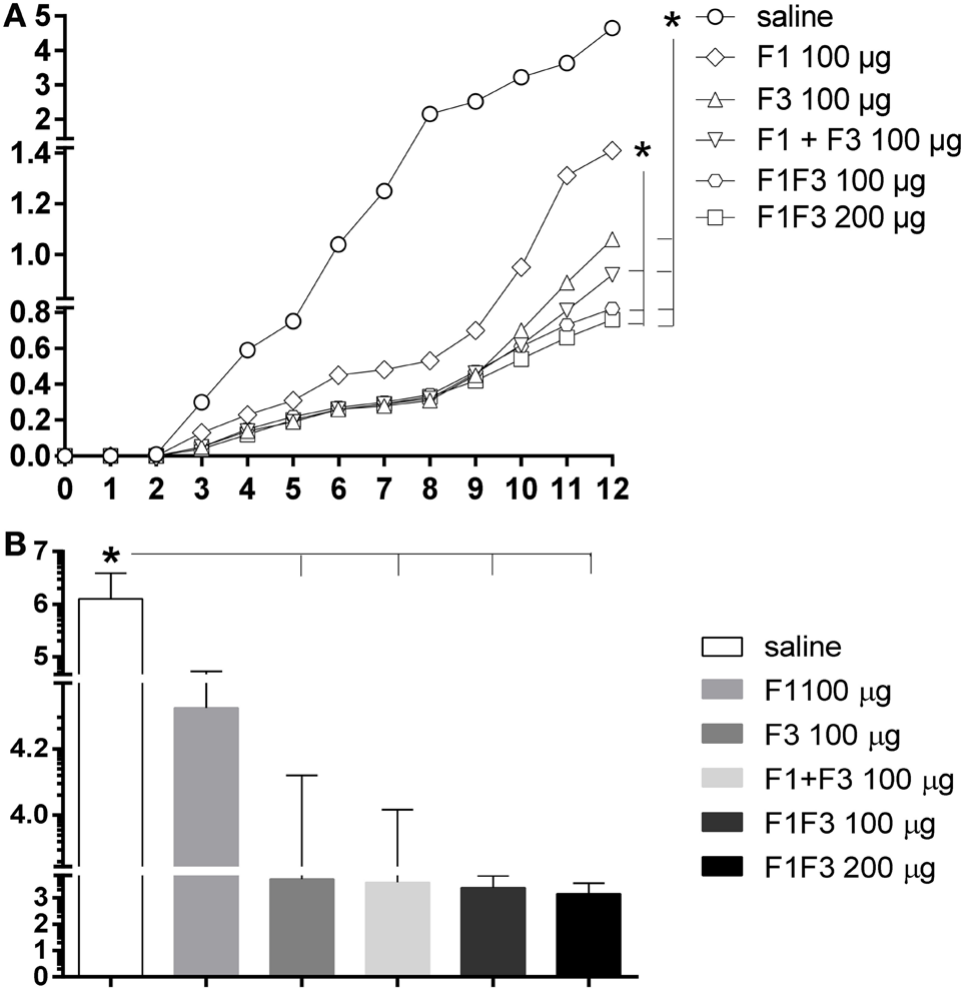
**Chimera optimized the vaccine efficacy by inducing the strongest reductions in sizes and parasite load of the skin lesions**. BALB/c mice were immunized with three subcutaneous doses of F1, F3, F1 and F3 (F1 + F3), or F1F3 chimera (100 or 200 μg) formulated in saponin and were challenged with *Leishmania amazonensis*. The evolution of footpad lesion sizes was weekly monitored with a pachymeter **(A)**. The values of the infected right hind-footpads were discounted from those of the contra-lateral left hind-footpads injected with saline. The parasite load in the skin lesion was measured after euthanasia by a limiting dilution assay on day 4 after *in vitro* culture **(B)**. Data represent means **(A)** and means + SE **(B)** of two independent experiments, each one of them with 8–10 animals per treatment. Except for the F1 vaccine, all formulations induced protection and decreased the lesion sizes in comparison to saline controls (linear regression analysis) **(A)**. The F1 + F3 vaccine and the chimeras were stronger than the F1 vaccine but, on week 12, however, the strongest reductions in lesion sizes were determined by the combination of F1 + F3 domains (80%) and by chimera at the 100 μg (82%) and 200 μg (84%) dosages **(A)**. The parasite load, on the other hand, revealed efficacies of 99.8 and 99.9% developed by the chimeras at 100 and 200 μg/dose, respectively **(B)**.

The evolution of the infection was monitored by the increase of the skin lesion sizes up to week 12 after infection. The linear regression analysis revealed significantly different degrees of vaccine-induced protection disclosed by the decreased slopes of their respective curves: 0.3033 ± 0.016 for the F1, 0.2257 ± 0.012 for the F3, 0.1973 ± 0.009 for the F1 + F3, 0.1793 ± 0.008 for the F1F3 100 μg, and 0.1640 ± 0.007 for the F1F3 200 μg vaccines. Except for the F1 vaccine, all formulations induced protection and decreased the lesion sizes in comparison to saline controls (*p* < 0.0351 for all comparisons) (Figure 7A). Furthermore, the F1 + F3 vaccine and the chimeras were more efficacious than the F1 vaccine (*p* < 0.0500) (Figure 7A) and equally potent until week 8 after infection. By the end of the experiment, on week 12, however, the strongest reduction in lesion sizes was determined by the combination of F1 + F3 domains and by the chimera, at the 100 and 200 μg dosages, which induced 80, 82, and 84% of protection, respectively (Figure 7A).

Additionally, the parasite load was evaluated after euthanasia by a limiting dilution assay on week 12 after infection (Figure 7B). While the saline controls exhibited 1,259,770 promastigotes, the chimera vaccines at 100 μg/dose decreased the parasite load to 2,423 (*p* < 0.0040) and at 200 μg/dose, to 1,424 (*p* < 0.0062). Our results indicate that the presentation of epitopes in tandem in a recombinant chimera exceeds the protection generated by the mixture of the recombinant domains F1 and F3. In fact, efficacies of 99.8 and 99.9% were achieved using the chimera at 100 and 200 μg/dose, respectively. The measures of footpad lesions at week 12 after infection and the log_10_ number of parasites in lesions were highly correlated (*p* < 0.001, *R* = 0.5640, *R*^2^ = 0.3181).

The increases in the antibody response were good surrogates for protection. In fact, we detected significant negative correlations between the increase of all antibody subtypes and the decrease of footpad lesion sizes and of the number of parasites. The IgG2a increase for instance was negatively correlated to the size of footpad lesions (*p* < 0.0001, *R* = −0.6026 before and *p* = 0.0029, *R* = −0.4246 after infection) and to the number of parasites in lesions (*p* = 0.0293, *R* = −0.3147 before and *p* = 0.0031, *R* = −0.4177 after infection).

Additionally, the IDR (*p* < 0.0001, *R* = −0.7815) and the frequencies of multifunctional CD4+ (*p* < 0.0500, *R* = −0.2803) and CD8+ T cells (*p* < 0.0001, *R* = −0.7837) expressing IL-2, TNF-α, and IFN-γ, after infection, were strong correlates of prophylactic efficacy. In agreement to that, the number of parasites in lesions was also negatively correlated with the IDR (*p* = 0.0012, *R* = −0.4531) and the CD8 T cells expressing IL-2, TNF-α, and IFN-γ (*p* = 0.0012, *R* = −0.4531).

We conclude that vaccination with the F1F3 chimera optimizes the cross-species vaccine efficacy against *L. amazonensis* infection above the levels reached by the admixed domains.

### CD4+ and CD8+ T-Cell Epitopes of the F1 and F3 Domains Exceed the Cellular Immune Response Generated by the Chimera

Toward the design of a potential synthetic vaccine, we further investigated which of the predicted F1 and F3 domains’ T-cell epitopes target the optimized cellular immune response and induce *in vitro* T cell responses even stronger than the chimera.

The *in silico* prediction for CD4+ T cell epitopes performed before (17) mapped two sequences of CD4+ T cell epitopes of BALB/c mice in F1 (ELLAITTVVGNQ and DVAGIVGVPVAAGCT) and three more sequences in the F3 domain (FMLQILDFYTKVYE, FRYPRPKHCHTQVA, and KFWCLVIDALKRIG) (Table 1). Additionally, the highest scored CD8+ epitope (YPPEFKTKL) of the NH36 protein was identified in the F1 domain (17) (Table 1). We here compared the secretion of cytokines induced by each one of these epitopes alone or mixed together, using the NH36 and F1F3 chimera antigens as controls, in mice vaccinated with the chimera, on week 11 after *L. amazonensis* challenge.

**Table 1:** **IFN-γ/IL-10 and TNF-α/IL-10 ratios secreted in response to the predicted synthetic epitopes of NH36**.

Amino acid location^a^	Predicted for	Domain	Sequences	IFN-γ/IL-10	TNF-α/IL-10
0219-40	CD4	F1	ELLAITTVVGNQ	0.18	0.81
54-68	CD4	F1	DVAGIVGVPVAAGCT	0.14	0.59
92-100	CD8	F1	YPPEFKTKL	0.96	0.49
217-230	CD4	F3	FMLQILDFYTKVYE	0.64	0.60
278-291	CD4	F3	FRYPRPKHCHTQ	0.92	1.72
298-311	CD4	F3	KFWCLVIDALKRIG	0.29	1.00

*^a^The *in silico* predicted epitopes for CD4+ and CD8+ lymphocytes of Balb/c mice were previously described by Nico et al. (17)*.

**Figure 8:**
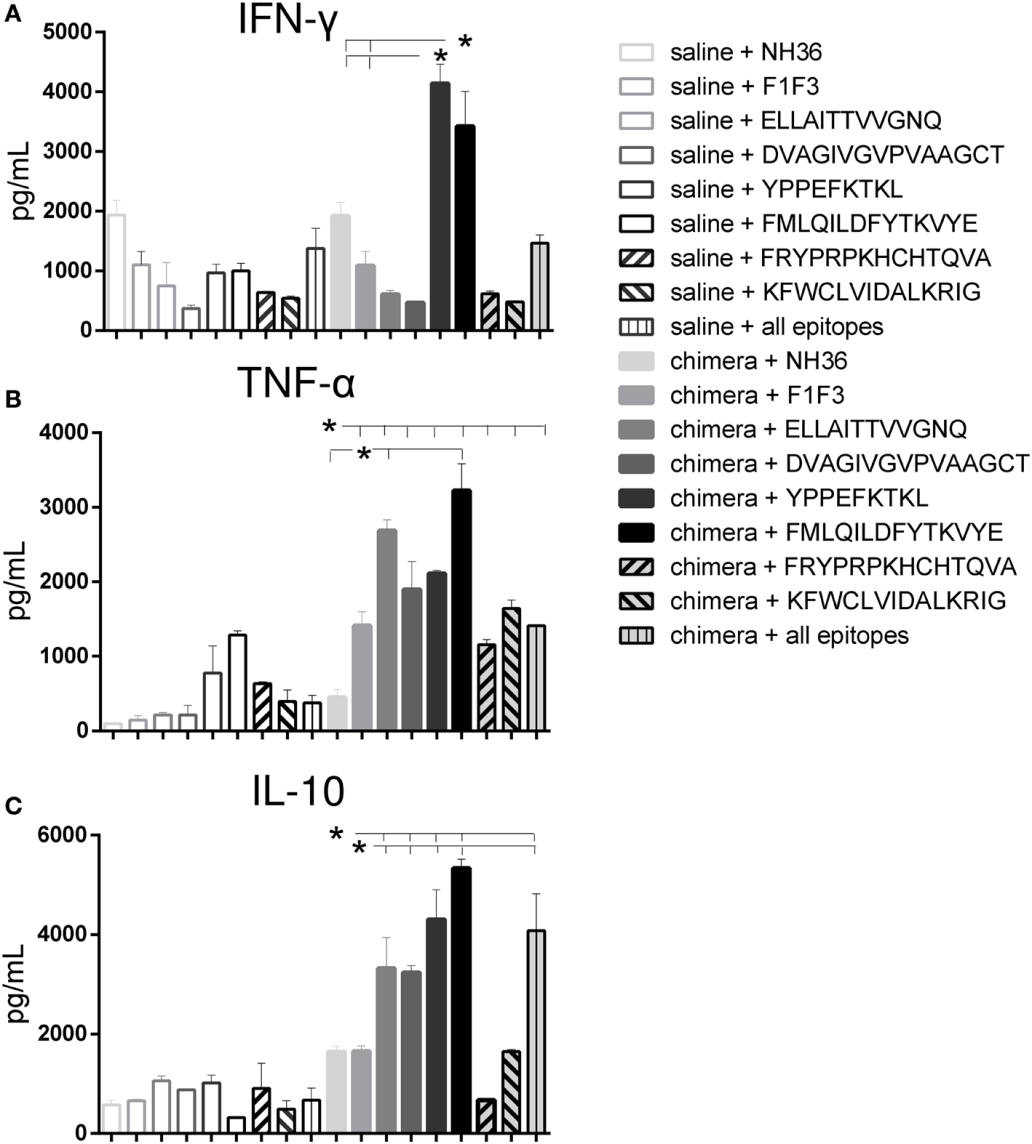
**The YPPEFKTKL and FMLQILDFYTKVYYE epitopes promote the highest IFN-γ and IL-10 levels, and together with DVAGIVGVPVAAGCT and ELLAITTVVGNQ, the strongest TNF-α secretion of splenocytes of chimera-vaccinated mice**. Splenocytes of mice vaccinated with 100 μg of the chimera were incubated *in vitro* with 10 μg/ml of NH36, F1F3 chimera, each one of the CD4 predicted epitopes of F1 (ELLAITTVVGNQ and DVAGIVGVPVAAGCT) and F3 domains (FMLQILDFYTKVYE, FRYPRPKHCCHTQVA, and KFWCLVIDALKRIG) and with the highest scored CD8 predicted epitope of the F1 protein (YPPEKTKL), or with the mixture of all the epitopes, at week 11 after infection. Secretions of IFN-γ **(A)**, TNF-α **(B)**, and IL-10 **(C)** were measured by an ELISA assay in the supernatants of splenocytes and expressed in picograms per milliliter. Data are means + SE of two independent experiments, each one with 8–10 animals per treatment. The YPPEFKTKL epitope of F1 and the FMLQILDFYTKVYE epitope of the F3 domain promoted the highest levels of IFN-γ **(A)**, and together with ELLAITTVVGNQ and DVAGIVGVPVAAGCT epitopes, also the strongest secretion of TNF-α **(B)** and IL-10 **(C)**. In contrast, the FRYPRPKHCHTQVA and KFWCLVIDALKRIG of F3 induced a moderate but significant secretion of TNF-α **(B)** and no secretion or poor levels of IL-10 **(C)**.

Actually, several epitopes induced higher cytokine secretion than the NH36 and their chimera cognate proteins in vaccinated mice and that their controls in saline treated mice. Among them, remarkably, the YPPEFKTKL epitope of F1 and the FMLQILDFYTKVYE epitope of the F3 domain promoted the highest levels of IFN-γ (Figure 8A), and together with ELLAITTVVGNQ and DVAGIVGVPVAAGCT epitopes, also the strongest secretion of TNF-α (Figure 8B) and IL-10 (Figure 8C).

In contrast, the two other CD4+ predicted epitopes of F3, FRYPRPKHCHTQVA and KFWCLVIDALKRIG, induced a moderate but significant secretion of TNF-α (Figure 8B) and no secretion or poor levels of IL-10 (Figure 8C), respectively, when compared to the untreated saline controls.

These results suggested the induction of a mixed Th1/Th2 immunity in response to the FMLQILDFYTKVYE of F3, and the YPPEFKTKL, ELLAITTVVGNQ, and DVAGIVGVPVAAGCT predicted epitopes of the F1 domain. In contrast, the two final CD4 predicted epitopes of F3, FRYPRPKHCHTQVA and KFWCLVIDALKRIG generated a main Th1 response, with a predominant TNF-α production and low IL-10 secretion (Figure 8).

Calculation of the IFN-γ/IL-10 and TNF-α/IL-10 secreted ratios confirmed the generation of the Th1 response (Table 1). In fact, the FRYPRPKHCHTQVA sequence of F3 induced an elevated IFN-γ/IL-10 and the highest TNF-α/IL-10 ratio. The KFWCLVIDALKRIG epitope also generated a high TNF-α/IL-10 ratio, which was followed by the ELLAITTVVGNQ sequence. Interestingly, the YPPEFKTKL epitope also promoted a high IFN-γ/IL-10 ratio (Table 1).

**Figure 9:**
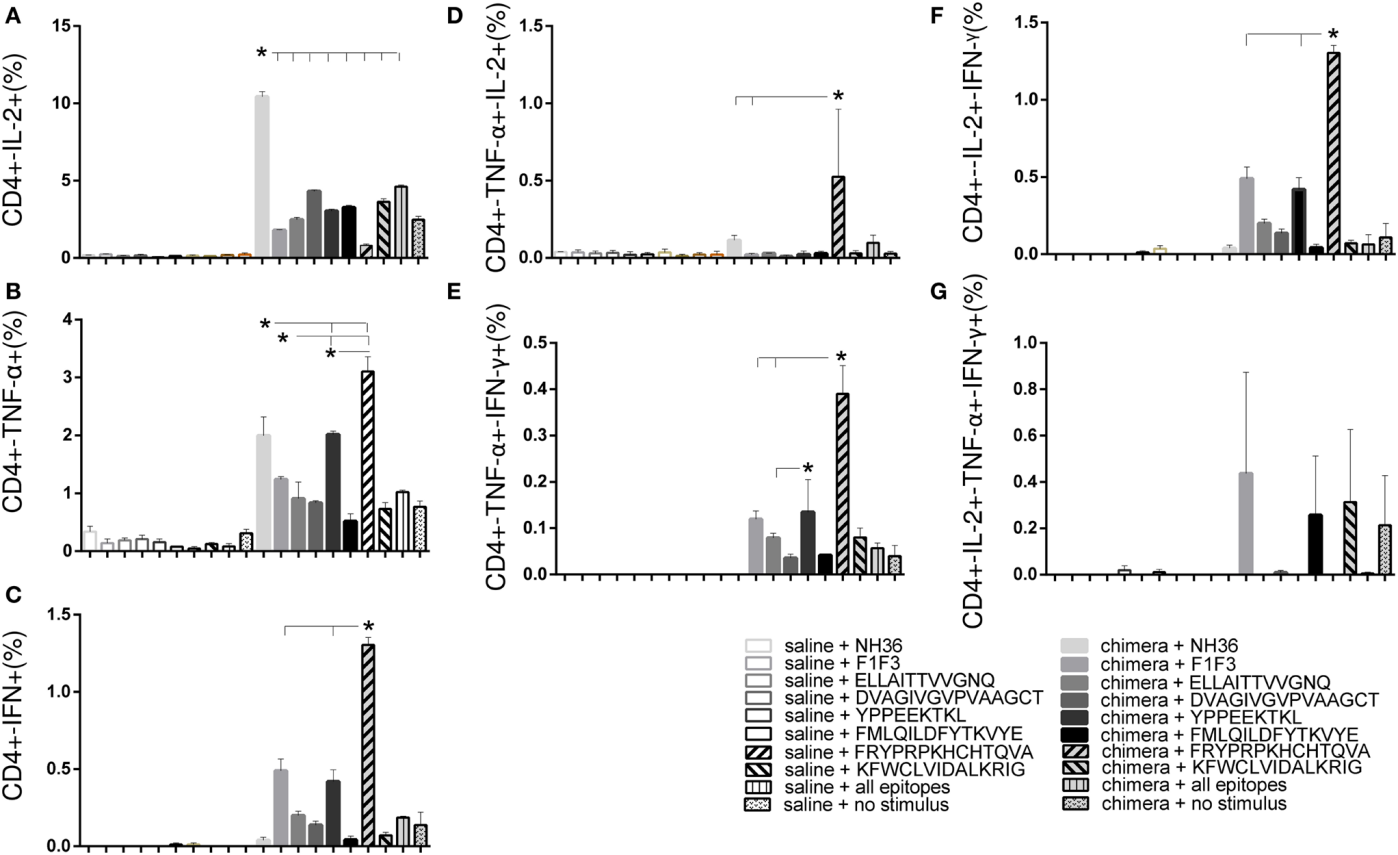
**Multiparameter cytometry analysis disclosed that the FRYPRPKHCHTQVA followed by the YPPEFKTKL epitopes induced the most potent CD4+ T cell response**. Splenocytes of chimera-vaccinated mice were incubated with NH36, the chimera, the ELLAITTVVGNQ, DVAGIVGVPVAAGCT, YPPEKTKL, FMLQILDFYTKVYE, FRYPRPKHCCHTQVA, and KFWCLVIDALKRIG sequences, or with the mixture of all the epitopes, at week 11 after infection. The magnitude of the CD4+ T cell response was disclosed by the frequencies of the CD4+ lymphocytes expressing IL-2 **(A)**, TNF-α **(B)**, IFN-γ **(C)**, IL-2/TNF-α **(D)**, TNF-α/IFN-γ **(E)**, IL-2/IFN-γ TNF-α/IFN-γ **(F)**, and IL-2/TNF-α/IFN-γ TNF-α/IFN-γ **(G)** in response to each antigen. Bars represent means + SE of two independent experiments, each one with 8–10 animals per treatment. The FRYPRPKHCHTQVA epitope induced maximal CD4+-TNF-α, -IFN-γ, -TNF-α-IL-2, -TNF-α-IFN-γ, and -IFN-γ-IL-2 T cell proportions, confirming its capability of raising a specific Th1 response. The YPPEFKTKL epitope was the second most important sequence to enhance the proportions of CD4+ T cells producing TNF-α, IFN-γ, IL-2-IFN-γ, and TNF-α-IFN-γ. The IL-2-TNF-α-IFN-γ-secreting CD4+ T-cells were only raised in response to the FRYPRPKHCHTQVA, FMLQILDFYTKVYE, and the admixed epitopes. In contrast, the chimera promoted the strongest CD4+-IL-2 T cell response.

The multiparameter cytometry analysis disclosed that the FRYPRPKHCHTQVA epitope as the most potent enhancer of the CD4+-TNF-α, -IFN-γ, -TNF-α-IL-2, -TNF-α-IFN-γ and -IFN-γ-IL-2 T cell proportions (Figure 9), confirming its capability of raising a specific Th1 response (Figures 8B,C). The YPPEFKTKL epitope was the second most important sequence which, although predicted as a CD8 epitope, also stimulated the increase of the proportions of CD4+ T cells producing TNF-α, IFN-γ, IL-2-IFN-γ and TNF-α-IFN-γ, as much as the chimera did (Figure 9). Additionally, the multifunctional IL-2-TNF-α-IFN-γ-secreting CD4+ T-cells were only raised in response to the FRYPRPKHCHTQVA, FMLQILDFYTKVYE, and the admixed epitopes. In contrast, no epitope increased the percent of CD4+ T cells secreting only IL-2 above the levels promoted by the chimera (Figure 9).

**Figure 10:**
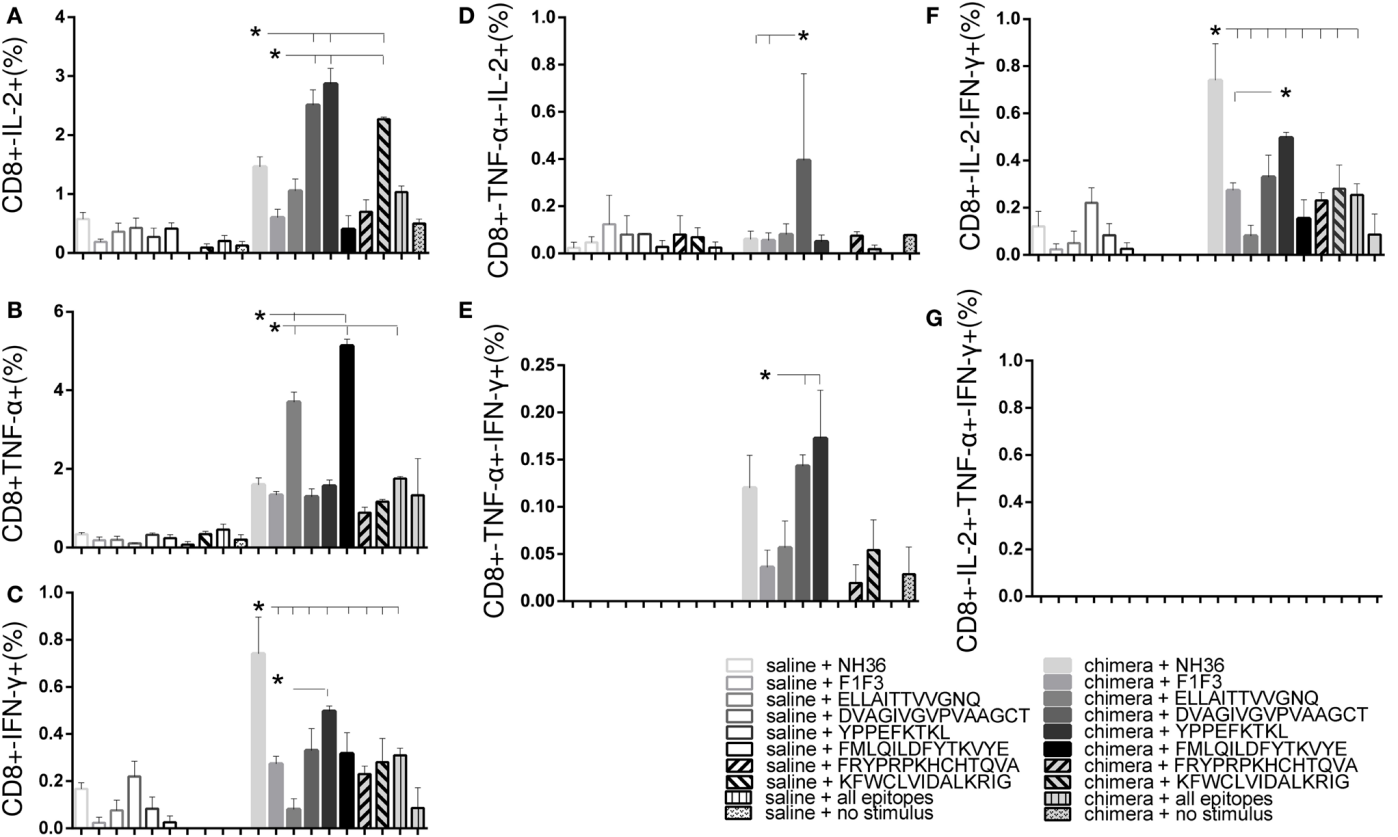
**Multiparameter cytometry analysis disclosed that the YPPEFKTKL followed by the DVAGIVGVPVAAGCT, FMLQILDFYTKVYE, and ELLAITTVVGNQ epitopes induced the most potent CD8+ T cell response**. Splenocytes of chimera-vaccinated mice were incubated with NH36, the chimera, the ELLAITTVVGNQ, DVAGIVGVPVAAGCT, YPPEKTKL, FMLQILDFYTKVYE, FRYPRPKHCCHTQVA, and KFWCLVIDALKRIG sequences, or with the mixture of all the epitopes, at week 11 after infection. The magnitude of the CD4+ T cell response was disclosed by the frequencies of the CD4+ lymphocytes expressing IL-2 **(A)**, TNF-α **(B)**, IFN-γ **(C)**, IL-2/TNF-α **(D)**, TNF-α/IFN-γ **(E)**, IL-2/IFN-γ TNF-α/IFN-γ **(F)**, and IL-2/TNF-α/IFN-γ TNF-α/IFN-γ **(G)** in response to each antigen. Bars represent means + SE of two independent experiments, each one with 8–10 animals per treatment. YPPEFKTKL was the most potent epitope. Alone, it induced higher proportions of CD8+ T cells secreting IFN-γ and IFN-γ in combination with IL-2 than the chimera; together with DVAGIVGVPAAGCT and KFWCLVIDALKRIG, the highest frequencies of CD8+-IL-2 T cells and combined only with DVAGIVGVPAAGCT, the highest proportions of CD8+-TNF-α-IFN-γ T cells. In addition, DVAGIVGVPAAGCT increased the frequencies of CD8+-TNF-α-IL-2 T cells. In contrast, ELLAITTVVGNQ and the FMLQILDFYTKVYE epitopes increased the frequencies of T cells secreting only TNF-α.

Regarding the cytotoxic response, YPPEFKTKL was the most potent epitope. Alone, it induced higher proportions of CD8+ T cells secreting IFN-γ and IFN-γ in combination with IL-2 than the chimera; together with DVAGIVGVPAAGCT and KFWCLVIDALKRIG, the highest frequencies of CD8+-IL-2+ T cells and combined only with DVAGIVGVPAAGCT, the highest proportions of CD8+-TNF-α+-IFN-γ+ T cells. Furthermore, DVAGIVGVPAAGCT was the only epitope to increase the frequencies of CD8+-TNF-α+-IL-2+ T cells (Figure 10). In contrast, the cytotoxic T cells secreting only TNF-α were increased in response to the ELLAITTVVGNQ and the FMLQILDFYTKVYE epitopes (Figure 10).

## Discussion

NH36 is an important phylogenetic marker, highly conserved among the species of the *Leishmania* genus. Thus, it became a strong candidate antigen for the development of a bivalent cross-protective vaccine against both visceral and CL (17, 28, 29, 38). We previously demonstrated that the F3 protein of NH36 hosts the immunodominant CD4+ epitopes necessary for protection against *L. chagasi* (17) and *L. amazonensis* (17, 28) infections. Instead, the F1 protein codominance with F3, in protection against *L. amazonensis* infection, is mediated mostly by CD8+ epitopes (28). Accordingly, the highest scored epitope for CD8+ T cells, YPPEFKTKL, was identified in the sequence of the F1 protein (17). The finding of 93% sequence homology between the NH36 of *L. donovani* and the A34480 NH of *L. amazonensis* encouraged even more the idea of a NH-based bivalent vaccine against both leishmaniasis (28).

According to the philosophy of the T cell polytope vaccine development, efficacy increases using antigens that contain enriched proportions of the relevant epitopes (34, 35). Searching for the optimization of the vaccine efficacy against *L. amazonensis* infection, in this investigation, we used the F1 and F3 domains of NH36 through several approaches. First, we investigated if the simple mixture of F1 and F3 vaccines increased the efficacy above the levels induced by the F1 and F3 vaccines independently. Second, we asked if the presentation of F1 and F3 domains cloned in tandem, as a chimera, was more efficacious than the presentation of the admixed proteins. Third, we assayed if the chimera-induced protection could be enhanced in a dose–response manner by doubling the vaccine concentration. Finally, we identified the most important epitopes for CD4+ and CD8+ T cells included in the chimera that could be combined in a future synthetic polytope vaccine capable to induce cross-protection to CL.

Confirming that the F3 and F1 domains contain the epitopes responsible for the NH36-induced immune protection, higher efficacy was obtained by vaccination with each one of these proteins rather than with the whole NH36 cognate protein (17, 28, 29, 33). Additionally, the cross-protective capabilities of the NH36 vaccines can be explained by the high identity of the sequences of predicted epitopes of the two *Leishmania* NHs (28). In fact, the CD4+ epitope ELLAITTVVGNQ and the CD8+ epitope YPPEFKTKL of the F1 domain of NH36 (17) are completely conserved in the sequence of NH A34480 of *L. amazonensis* (28). Likewise, the DVAGIVGVPVAAGCT epitope for CD4+ T cells of F1, and the three epitopes for CD4+ T cells of F3, FMLQILDFYTKVYE, FRYPRPKHCHTQVA, and KFWCLVIDALKRIG differ in only one amino acid (28).

Strong IgG1, IgG2a, and/or IgG2b anti-NH36 antibody responses were described after mice vaccination with the gene of NH36 (23, 24, 27), with the NH36 recombinant protein in combination with saponin (17, 28, 29), CPG and polylactil glycolide particles (39), and the GLA-SE adjuvant (40), or with the NH36 cloned with a sterol 24-C-methyl transferase (NS), and used with GLA-SE adjuvant (30). Furthermore, higher IgG2/IgG1 antibody ratios were also found after immunotherapy of *Leishmania infantum chagasi*-infected dogs with NH36-DNA (25). Additionally, human vaccination with NS determined a preferential increase in IgG1 and IgG3 subclasses, which are dependent of Th1-like cytokines (30).

Vaccination with F3, but not with F1, induced higher IgG2a antibody levels than NH36, in mice challenged with *L. chagasi* (17). In contrast, IgG2a, IgG1 antibody levels were similar in mice challenged with *L. amazonensis* (28, 29). Although protection against leishmaniasis depends more, on the cellular than on the humoral immune response, it is worth to note that these increased antibody subtypes are strong surrogates predictive of protection (41), which indicate the decrease of parasite load (17, 28, 29) and that could also be the basis of the development of a transmission blocking vaccine (38, 42).

We previously identified in the F3 domain, the epitopes AVQKRVKEVGTKPAAFML (202–219), VYEKERNTYATV (228–239), and FRYPRPKHCHTQVA (278–291), as the most relevant targets of the anti-NH36 antibody response in mice (17). Supporting our results, two B cell epitopes for dog and human antibodies were confirmed in the sequence of NH36 by other group (43). These epitopes, called peptide 17 and 18 (43), overlap with the sequences AVQKRVKEVGTKPAAFML and NQTLEKVTRNARLVADVAG that we previously described in the F3 and F1 domains, respectively (17). Interestingly, the peptide17 diagnosed with 100% sensitivity canine and human VL (43). These results disclose the universal nature of the B cell epitopes of NH36.

We further demonstrated that the admixed F1 + F3 vaccine induced more IgG, IgG1, and IgG2a antibodies than the F3 or the F1 vaccines did independently, suggesting a potentiated immunogenicity. However, the presentation of the domains cloned in tandem as the chimera was superior and exceeded the IgG and IgG2a response above the admixed domains showing an additional dose–response effect.

Our results demonstrate that the delivery of the F1 and F3 domains cloned in tandem, rather than as a simple mixture, increases the probabilities of a single antigen presenting cell to exhibit simultaneously both the CD8+ and CD4+ epitopes to lymphocytes, optimizing the immunogenicity. This might occur through cross-priming when MHC class II molecules can present both, exogenous molecules acquired by antigen presenting cells and endogenous degraded antigens (44, 45). Therefore, our results suggest that not only the composition of the epitopes and subunits is critical but also how the epitopes are exposed in the tridimensional structure of the vaccine antigen (46). In fact, only the tandem arrangement, but not the independent subunits containing an HIV epitope represented the most efficient immunogenic conformation (46). Another advantage of the presentation of the epitopes in a recombinant chimera is that it facilitates the scaling-up of the antigen, reducing the production yield time and cost, when large amounts of antigen for clinical phase III and IV assays are needed.

Regarding the generation of the cellular immune response, the F3 domain induced stronger IDR than the F1 protein in mice vaccinated against *L. chagasi* (17) and *L. amazonensis* (28, 29). In this investigation, we showed that the admixed antigens were even better than F3, but also, that the chimera induced the strongest IDR after infection and an increased a dose–response effect. Supporting our previous descriptions (17, 28, 29), the IDR was an important correlate of protection that increased along with the decrease of parasite load, indicating the generation of a robust cellular immune response against *L. amazonensis* optimized by vaccination with the chimera.

We confirmed here our previous results showing that mice vaccinated with F3 and challenged with *L. amazonensis* increase the IFN-γ and TNF-α secretion but have a null IL-10 response (28, 29). Mice vaccinated with the F1 domain, on the other hand, secreted IFN-γ, TNF-α, and IL-10, suggesting the simultaneous stimulation of T reg subsets and the presence of epitopes for T regs along its sequence (28, 29). In this investigation, vaccination with the chimera induced a concomitant higher expression of IFN-γ and IL-10 than the admixed domains, and a dose–response effect. This is remarkable considering that IL-10 has been shown to be related both to the pathology and the control of CL (47) and that IFN-γ has also a dual role, as inducer of effector mechanisms and, conversely, as a mediator of inflammation and pathogenesis (48). Increased CD4+ T cell proportions secreting IFN-γ with low proportions of CD4+-IL-4 T cells were also described in mice vaccinated with NH36 and challenged with *L. mexicana* (40).

We additionally demonstrated that the chimera vaccine at 100 μg/dose induced the highest proportions of all types of CD4+ and CD8+ T cells secreting IL-2, TNF-α, or IFN-γ alone, TNF-α in combination with IL-2 or IFN-γ, and the highest percentage of CD4+ multifunctional cells secreting IL-2, TNF-α, and IFN-γ simultaneously. In fact, the chimera optimized the induction of a CD4+ Th1 immune response by promoting the highest proportions of CD4+ effector and long-term memory potential T cells, being the strongest vaccine at all the steps of the CD4 T cell differentiation (36). Thus, and confirming the *in silico* prediction (17) important epitopes for CD4+ T cells are present both in the F1 and F3 domains and their presentation in tandem, in the chimera, improve significantly their immunogenicity. This very desirable performance was observed also for the cytotoxic immune response. The highest proportions of all subtypes of CD8+-cytokine secreting T cells were observed in response to the chimera, except for the multifunctional cells. As detected for the CD4+ T cells, F1 was the second most important vaccine for the induction of the cytotoxic response while no enhancement was induced by the admixed antigens.

The immunotherapeutic effect induced by the F1 or F3 domains against *L. amazonensis* infection was predicted by the frequencies of the CD4+ and CD8+ T cells producing IL-2 or TNF-α or both (29). Total frequencies and frequencies of double-cytokine CD4 T cell producers were enhanced by F1 and F3 vaccines. In contrast to what described for prophylactic vaccination with the chimera, no increases in CD4+ multifunctional T cells were observed after immunotherapy with the independent domains (29). On the other, the F1 vaccine was codominant in prophylaxis with the chimera, for both CD4+ and CD8+ T cells (this investigation) and promoted the highest proportions of CD8+ multifunctional T cells when used for immunotherapy (29).

The reduction of parasite load and sizes of lesions confirmed the predictions of the immunological assays. Cross-protection to *L. amazonensis* infection was maximal (99.8–99.9%) in mice vaccinated with the chimera. As described before, and in spite of raising a strong CD4+ and CD8+ T cell immunogenic response, the F1 vaccine was less protective (17, 28).

We concluded that the chimera optimized the immune cross-protection against *L. amazonensis*, above the levels induced by the F1 and F3 domains either admixed or independently. Our results indicated the development of a Th1-immune response mediated by CD4+ T cell epitopes of the F3 and F1 domains, a cytotoxic response induced by F1 and the potential presence of T reg epitopes in the F1 domain determining also a potential regulatory response. The three arms of immunity increased by the presentation of the F1 and F3 epitopes in tandem in the F1F3 chimera.

Progressing toward the definition of a polytope vaccine, we were further able to identify the epitopes of NH36 responsible for the cross-protection against *L. amazonensis* infection. Confirming that they are the target of the immune response, some epitopes exceeded the levels of cytokine secretion induced by the chimera (33). The FMLQILDFYTKVYE and YPPEFKTKL sequences promoted, respectively, a 3.2- and 3.8-fold enhance in IFN-γ, a 3.2- and 2.9-fold increase in IL-10, and a 2.3- and 1.5-fold augment of TNF-α secretion above the levels generated by the chimera.

Additionally, we further elucidated that the main Th1 response induced by the NH36 and the chimera was due to the FRYPRPKHCHTQVA epitope of F3, which generated elevated IFN-γ/IL-10 and TNF-α/IL-10 ratios and the highest proportions of CD4+-TNF-α, -TNF-α-IL-2, -TNF-α-IFN-γ, -IFN-γ-IL-2, and multifunctional IL-2-TNF-α-IFN-γ T cells. The FRYPRPKHCHTQVA epitope might be the reason for the CD4+ Th1 TNF-α-mediated protection induced by F3 and previously detected against both visceral (17) and CL in mice [(this investigation) (28, 29)]. In fact, only the FRYPRPKHCHTQVA sequence determined a high CD4+ response with strong IFN-γ, TNF-α secretion, and null IL-10 response, as already described for the F3 vaccine (17, 29).

Although first predicted as a CD8 epitope (17, 28), the YPPEFKTKL sequence of F1 also contributed to the CD4+ T cell response. In fact, it was the second most important CD4+-epitope, which stimulated a high IFN-γ/IL-10 ratio and increased proportions of CD4+ T cells producing TNF-α, IFN-γ, IL-2-IFN-γ, and TNF-α-IFN-γ. Additionally, the YPPEFKTKL and DVAGIVGVPAAGCT epitopes were the most important sequences inducing the CD8+ T cell response. Likewise, the ELLAITTVVGNQ and the FMLQILDFYTKVYE epitopes also induced elevated frequencies of CD8−TNF-α T cells, although they were predicted as CD4+ T cell epitopes of mice (17, 28). In agreement with that, the AFMLQILDF sequence was also predicted as a human epitope of CD8+ T cells binding the HLA-A*2402 and HLA-B*4402, HLA-A*01 molecules (49).

We demonstrated that the YPPEFKTKL epitope capabilities of stimulating both the CD4+ and CD8+ T cell response, with intense production of pro-inflammatory cytokines, indicating its potential PAN epitope nature (50). YPPEFKTKL was the only predicted epitope for CD8+ T cells identified in the F1 domain (17) and because of that, it is probably the responsible for the CD8−T cell mediate vaccine protection against *L. amazonensis* infection, attributed to the F1 vaccine (28). The enhancement of the IL-10 secretion induced by the F1 vaccine [(this investigation) (28, 29)] and YPPEFKTKL indicate that this sequence might also be a T regulatory epitope, which deserves better characterization. Supporting its universality and biological relevance, the YPPEFKTKL sequence was also predicted with high scores for the binding of the human Class I HLA-A*2402 and HLA-B*0702 molecules, induced the IFN-γ secretion by PBMC of asymptomatic, IDR positive human individuals infected with *L. infantum* and was found to be highly conserved in the NH sequence of all the studied *Leishmania* species (49).

For its multiple capabilities, the YPPEFKTKL epitope might be considered as a Pan epitope candidate (51–53). In fact, the sequence MDEPTLLYV was described as a PANDR epitope of the A15 hexon protein of adenovirus (52) while the synthetic more preferably PADRE peptide composition is aKXVAAWTLKAAa (54). In order to constitute a PADRE epitope, the peptide should contain defined amino acids in R1, R2, R3, R4, and R5 (54). Remarkably, and as expected for a PADRE sequence (54), the YPPEFKTKL contains Y as the R2 residue, four residues in R3, where three to five amino acids are needed, and the sequences KT followed by TKL in R4, while the expected combinations for R4 are KT, TLK, or WTLK (54). The adenovirus epitope contains TLL instead of TLK in the place of R4 (52).

YPPEFKTKL of *L. donovani* maintains identical sequence in the NHs of *L. infantum chagasi, L. infantum, L. amazonensis, L. major*, and *L. mexicana* (49). TKL is substituted with TNL in *L. tropica* while the second residue of R2, P, is substituted by S in the NHs of *L. braziliensis* and *Leishmania panamensis*, both of the subgenus *Vianna* (49).

Another common feature of the NH36 epitopes to PADRE sequences is found in the KFWCLVIDALKRIG CD4+ predicted epitope of F3, which shares with the aKFVAAWTLKAAa of the PADRE sequence the first KF residues (54). This epitope induced a mild secretion of IFN-γ, TNF-α, and IL-10 and increased frequencies of CD4+ multifunctional T cells.

In spite the intense research on development of anti-*Leishmania* vaccines in animals models (11, 55) only a few of them are the basis of potential synthetic or epitope vaccines. The kmp-11 (56), the amastigote A2 (57), and the polytope vaccine containing LPG-3, LmSTI-1, CPB, and CPC (58) present epitopes for the CD8+ T cells. On the other hand, the LACK158–173 peptide (59), the amastigote A2 antigen (57), and the MML-triple fusion *L. major* vaccine expressed in adenovirus (37) determined a Th1-biased CD4 T cell response.

In this investigation, we demonstrated that arrangement of the epitopes of the NH of *L. donovani* NH36 in tandem, in a chimera, improved the cross-protection to CL caused by *L. amazonensis*. We found that the chimera optimized the vaccine efficacy, probably by increasing the probabilities of cross-presentation as a strategy to enhance immunity (44, 45). We further advanced in the identification of the most relevant epitopes responsible for the immune responses generated by the subunit vaccines (17, 28, 29). We found potent epitopes for the generation of the CD4+-Th1, cytotoxic and potential T regulatory response and recognized among them at least one with PADRE capabilities (54). The data gathered in our work will help in the development of a polytope vaccine against visceral and CL, which will allow to fight the disease with enhanced efficacy.

## Author Contributions

MA-S and DN conducted the experiments, MA-S, DN and AM acquired data, MA-S, DN, MP, and CP-d-S analyzed data. CP-d-S designed research studies. MA-S and CP-d-S prepared the figures. CP-d-S wrote the manuscript, and all authors have read and approved the final manuscript.

## Conflict of Interest Statement

DN, MP, and CP-d-S are inventors of the patent file PI1015788-3 (INPI Brazil). MA-S and AM declares no conflict of interest. The reviewer LFL declared a shared affiliation, though no other collaboration, with the authors to the handling Editor, who ensured that the process nevertheless met the standards of a fair and objective review.

## Footnotes

^1^http://bimas.dcrt.nih.gov/molbio/hla_bind/

^2^http://www.syfpeithi.de/

## Supplementary Material

Figure S1 **Monomer of *Leishmania donovani* nucleoside hydrolase (NH36)**. **(A)** Illustration of the tridimensional structure of the NH36 monomer was obtained by homology modeling to the sequence of the NH of *Leishmania major* template (RCSB PDB code: 1EZR; crystal structure of NH of *L. major*) with the sequences of the N-terminal (F1, amino acids 1–103 in lime green), central (F2, amino acids 104–198 in gray), and C-terminal (F3, amino acids 199–314 in cyan) moieties, using the Modeller 9.10 software. **(B)** MHC class II-IAd and IEd, haplotype H2d CD4+ T cell epitopes (dark blue) and of MHC class I Ld-CD8+ T cell predicted epitopes (red) of the C-terminal and N-terminal moieties. This illustration was modified from Nico et al. (29) and reproduced with authorization of the authors.
