# Supplementary figures and images for: A Chimera Containing CD4+ and CD8+ T-Cell Epitopes of the Leishmania donovani Nucleoside Hydrolase (NH36) Optimizes Cross-Protection against Leishmania amazonesis Infection
Source: Front Immunol. 2017 Feb 23;8:100. doi: 10.3389/fimmu.2017.00100 (PMC5322207; doi:10.3389/fimmu.2017.00100)

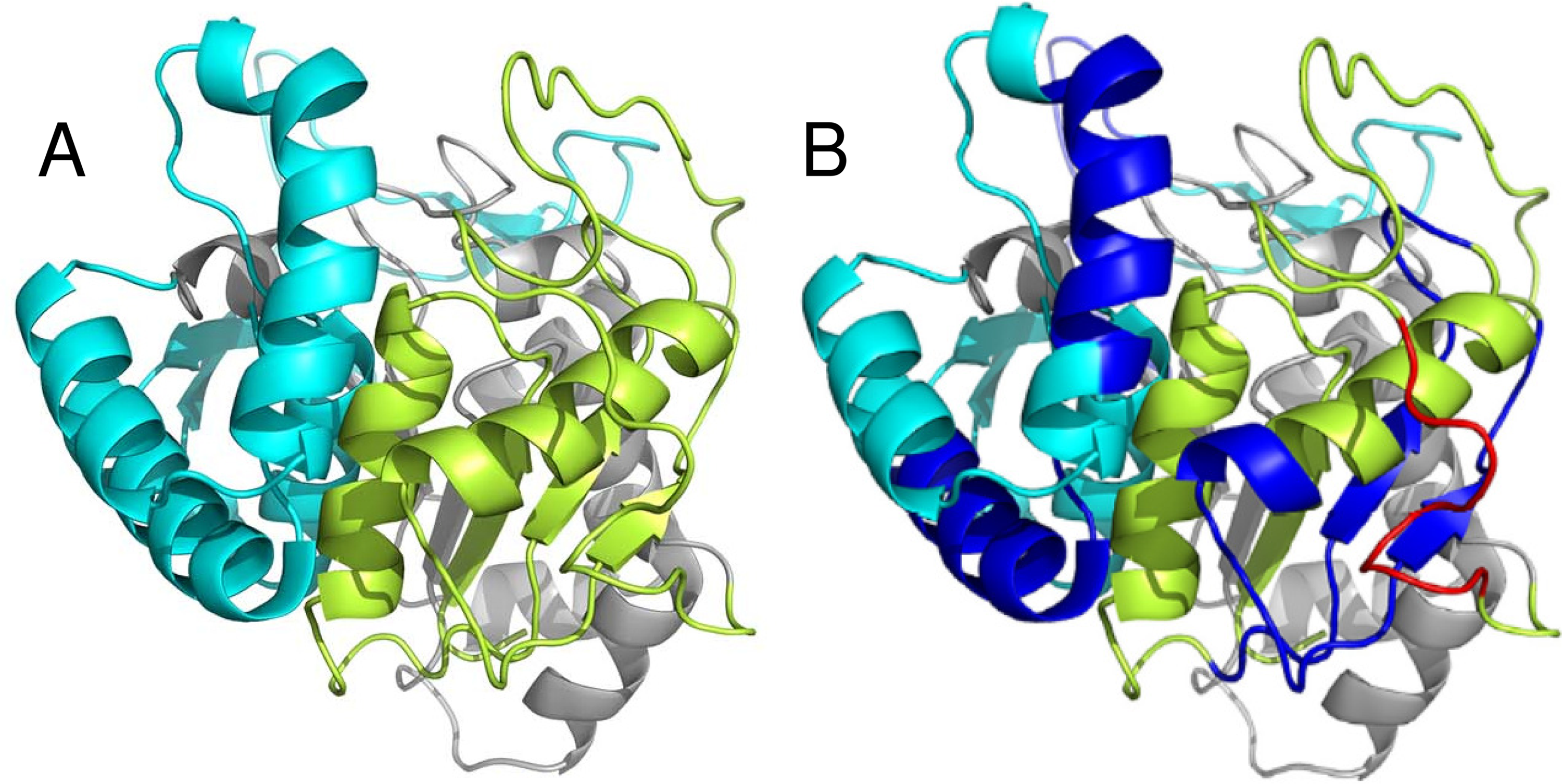

Supplement: Figure S1 — Monomer of Leishmania donovani nucleoside hydrolase (NH36). (A) Illustration of the tridimensional structure of the NH36 monomer was obtained by homology modeling to the sequence of the NH of Leishmania major template (RCSB PDB code: 1EZR; crystal structure of NH of L. major) with the sequences of the N-terminal (F1, amino acids 1–103 in lime green), central (F2, amino acids 104–198 in gray), and C-terminal (F3, amino acids 199–314 in cyan) moieties, using the Modeller 9.10 software. (B) MHC class II-IAd and IEd, haplotype H2d CD4+ T cell epitopes (dark blue) and of MHC class I Ld-CD8+ T cell predicted epitopes (red) of the C-terminal and N-terminal moieties. This illustration was modified from Nico et al. (29) and reproduced with authorization of the authors. [file Image_1.TIF]
